# Specificity of TGF-β1 signal designated by LRRC33 and integrin α_V_β_8_

**DOI:** 10.1038/s41467-022-32655-9

**Published:** 2022-08-25

**Authors:** Zelin Duan, Xuezhen Lin, Lixia Wang, Qiuxin Zhen, Yuefeng Jiang, Chuxin Chen, Jing Yang, Chia-Hsueh Lee, Yan Qin, Ying Li, Bo Zhao, Jianchuan Wang, Zhe Zhang

**Affiliations:** 1 State Key Laboratory of Membrane Biology, Center for Life Sciences, School of Life Sciences, Peking University, 100871 Beijing, China; 2 Molecular Cancer Research Center, School of Medicine, Shenzhen Campus of Sun Yat-sen University, No. 66, Gongchang Road, Guangming District, 518107 Shenzhen, Guangdong China; 3 Department of Structural Biology, St. Jude Children’s Research Hospital, Memphis, TN 38105 USA; 4 Parthenon Therapeutics, 40 Guest street, Boston, MA 02135 USA; 5 Center for Translational Research, Shenzhen Bay Laboratory, 518007 Shenzhen, Guangdong China

**Keywords:** Growth factor signalling, Cryoelectron microscopy, Integrins

## Abstract

Myeloid lineage cells present the latent form of transforming growth factor-β1 (L-TGF-β1) to the membrane using an anchor protein LRRC33. Integrin α_V_β_8_ activates extracellular L-TGF-β1 to trigger the downstream signaling functions. However, the mechanism designating the specificity of TGF-β1 presentation and activation remains incompletely understood. Here, we report cryo-EM structures of human L-TGF-β1/LRRC33 and integrin α_V_β_8_/L-TGF-β1 complexes. Combined with biochemical and cell-based analyses, we demonstrate that LRRC33 only presents L-TGF-β1 but not the -β2 or -β3 isoforms due to difference of key residues on the growth factor domains. Moreover, we reveal a 2:2 binding mode of integrin α_V_β_8_ and L-TGF-β1, which shows higher avidity and more efficient L-TGF-β1 activation than previously reported 1:2 binding mode. We also uncover that the disulfide-linked loop of the integrin subunit β_8_ determines its exquisite affinity to L-TGF-β1. Together, our findings provide important insights into the specificity of TGF-β1 signaling achieved by LRRC33 and integrin α_V_β_8_.

## Abstract (web summary)

Microenvironment localization and activation of L-TGF-β1 determine its specific function. Here, the authors elucidated the underlying mechanisms of specific presentation of L-TGF-β1 on the surface of myeloid lineage cells designated by LRRC33, and its activation by integrin αVβ8.

## Introduction

Transforming growth factor-β (TGF-β) is a pleiotropic cytokine with indispensable roles in extensive physiological processes such as organ development, immune response, and tissue homeostasis^1–4^. Dysregulation of TGF-β signaling is associated with many pathological conditions, e.g., cancer, autoimmune disease, fibrosis, and neurodegeneration^5–7^. Therefore, in-depth knowledge of TGF-β signaling mechanism is vital for understanding the body’s health and disease.

TGF-β is synthesized as a latent form (L-TGF-β) containing a 25-kDa N-terminal latency-associated peptide (LAP) and a 12-kDa C-terminal growth factor (mTGF-β) domain. L-TGF-β is folded as a homodimer in the endoplasmic reticulum and cleaved between LAP and mTGF-β by furin or furin-type proteases in the trans-Golgi network or the extracellular matrix^8^. Notably, LAP remains non-covalently associated with mTGF-β after cleavage, which prevents mTGF-β from binding to TGF-β receptors^8,9^. During biosynthesis, LAP is often covalently linked with specific anchor proteins through disulfide bonds. One class of such anchor proteins are latent TGF-β binding proteins (LTBPs), which deposit L-TGF-β to the extracellular matrix^10,11^. Additionally, two leucine-rich repeat-containing anchor proteins, LRRC32 (also known as GARP) and LRRC33 (NRROS), present L-TGF-β on the plasma membrane of regulatory T (T_reg_) cells and myeloid lineage cells (e.g., macrophages, dendritic cells, and microglia), respectively^12–14^. Therefore, specific presentation and storage of L-TGF-β in different extracellular microenvironment by different anchor proteins serve as a critical regulatory mechanism of TGF-β signal in various biological contexts.

mTGF-β must be discharged from the LAP-anchor protein complex to activate the downstream signaling pathway, and multiple factors can trigger this activation process^8^. α_V_β_6_ and α_V_β_8_ integrins are the most well-established activators for L-TGF-β, which bind to the RGDLXX(L/I) motif of LAP with nanomolar affinity^15–17^. Integrin α_V_β_6_ assists mTGF-β release by exerting tensile force to unfold LAP, and the attachment of L-TGF-β to the extracellular matrix or the cell surface by an anchor protein is required for such α_V_β_6_-mediated activation^8,15^. In contrast, the mechanism of activation by integrin α_V_β_8_ is still obscure. α_V_β_8_’s lower affinity to L-TGF-β (~50-fold lower than that of α_V_β_6_) and the absence of cytoskeleton attachment by the β8 leg both suggest that α_V_β_8_ is unable to transmit tensile force like α_V_β_6_. Conformational changes of the L-TGF-β-anchor protein complex induced by α_V_β_8_ binding likely facilitate other events leading to mTGF-β release^18,19^.

Despite great scientific advances achieved in the TGF-β field, the underlying mechanism designating the specificity of TGF-β signal remains incompletely understood. In particular, three closely related isoforms of TGF-β (i.e., TGF-β1, -β2, and -β3) are encoded in mammals. The growth factors of the three isoforms share 68% sequential identity and high structural similarity. These isoforms initiate essentially the same downstream signaling pathway, but they exhibit significantly different functions in physiology and disease^1,20–22^. How is such functional distinction achieved remains to be determined.

In this work, we focus on the mechanism of specific L-TGF-β1 presentation and activation mediated by LRRC33 and integrin α_V_β_8_ in myeloid lineage cells. We report cryo-EM structure of the human L-TGF-β1/LRRC33 complex and demonstrate that LRRC33 can presents only L-TGF-β1 but not the -β2 or -β3 isoforms due to differences of key residues on their growth factor domains. Moreover, we uncover a specific binding mode between integrin α_V_β_8_ and L-TGF-β1 by solving a cryo-EM structure of 2:2 human integrin α_V_β_8_/L-TGF-β1 complex which exhibits higher binding avidity and more efficient L-TGF-β1 activation. Finally, we reveal that the disulfide-linked loop (DLL) of the integrin subunit β_8_ determines its exquisite affinity to L-TGF-β1. These results offer crucial insights into the mechanism underlying the specificity of TGF-β1 signaling.

## Results

### Structure of the human L-TGF-β1/LRRC33 complex

**Fig. 1:**
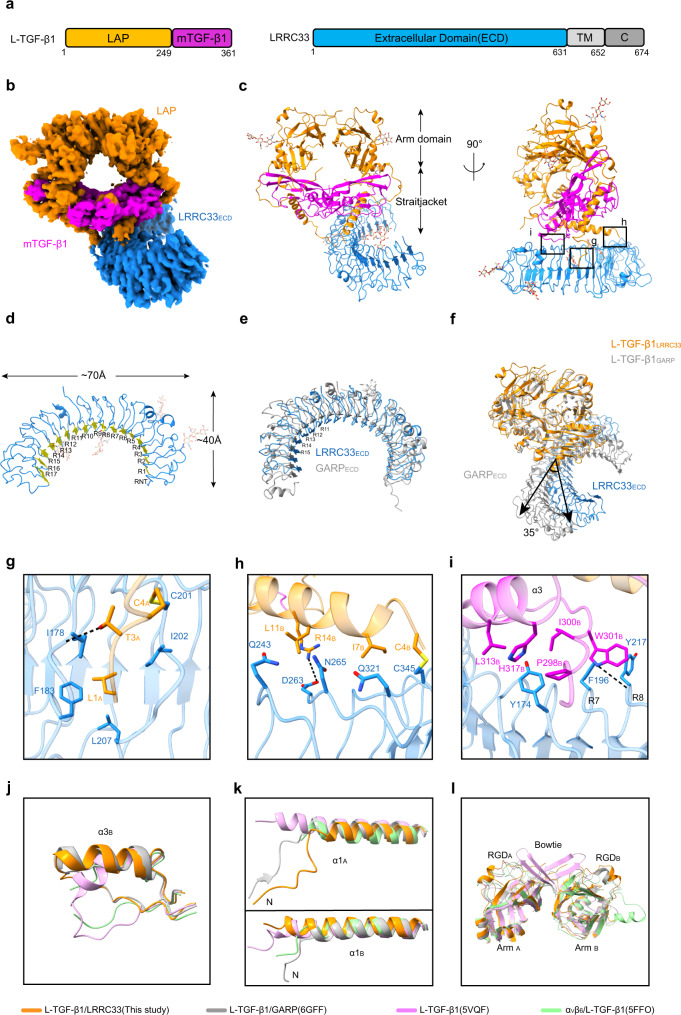
Cryo-EM structure of the L-TGF-β1/LRRC33 complex. **a** Sequence schematic diagrams of L-TGF-β1 and LRRC33. Chromatic bricks represent the protein regions used in our study, whereas gray bricks represent regions that are not included. Latency-associated peptide (LAP), mature TGF-β1 (mTGF-β1), and the extracellular domain of LRRC33 (LRRC33_ECD_) are colored in orange, magenta, and blue, respectively. **b** Cryo-EM map of the L-TGF-β1/LRRC33 complex. The contour level is 0.286. Individual domains are colored the same as those in **a**. **c** Overall structure of the L-TGF-β1/LRRC33 complex in ribbon presentation. The color code is the same as before. The Arm and straitjacket domains are labeled. **d** Ribbon diagram of LRRC33. R1 to R17, leucine-rich repeat (LRR); RNT, LRR N-terminal region. Yellow arrows represent the β strands in each repeat. **e** Superposition of individual LRRC33 and GARP (PDB code: 6GFF) from their complex structures with L-TGF-β1. LRRC33 is colored in blue, and GARP is colored in gray. R11-R15 of LRRC33 are indicated. **f** Superposition of L-TGF-β1/LRRC33 and L-TGF-β1/GARP (PDB code: 6GFF) complexes according to L-TGF-β1. Compared with GARP, LRRC33 rotates about 35 degrees relative to the diad axis of L-TGF-β1 dimer. **g**–**i** Interaction details between L-TGF-β1 and LRRC33 as indicated in the insets of **c**. Residues in different L-TGF-β1 monomers are distinguished by subscripts A and B. Black dashed lines represent hydrogen bond interaction (<4 Å). **j**–**l** Comparison of three distinguishing regions in L-TGF-β1 between our structure and others.

Human full-length L-TGF-β1 (i.e., residues 1–361) and LRRC33 ectodomain (i.e., residues 1–631) were co-expressed in Expi293F cells (Fig. 1a). The secreted L-TGF-β1/LRRC33 complex was purified for cryo-EM analysis. The best subset of particles could be refined to 4.01-Å resolution (Fig. 1b, Supplementary Figs. 1, 2, and Table 1). Aided by the reported structures of L-TGF-β1^9,23^ and L-TGF-β1/GARP^24^, we built the structure model for the L-TGF-β1/LRRC33 complex (Fig. 1c). Both L-TGF-β1 and LRRC33 have abundant N-linked glycosylation, which helps to validate this structural model (Supplementary Fig. 2e).

The LRR N-terminal region (RNT, residues 22–39) and seventeen LRR fragments (R1–R17, residues 40–501) of LRRC33 were mapped in the model (Fig. 1d). EM densities of the rest portion of LRRC33 appear discrete, implying the flexible nature of LRRs. LRRC33 adopts a classic solenoid structure of the leucine-rich repeat (LRR)-containing proteins. Superposition of LRRC33 and GARP shows a root-mean-square deviation (RMSD) of 3.89 Å for 309 Cα atoms^24^. In particular, the central region (R11-R15) of LRRC33 has an inward 3–5 Å movement (Fig. 1e). In addition, LRRC33 exhibits a 35-degree rotation compared to GARP^24^ when bound to L-TGF-β1 (Fig. 1f).

L-TGF-β1 takes similar global structures in our L-TGF-β1/LRRC33 complex and the reported L-TGF-β1/GARP complex^24^ (Fig. 1f and Supplementary Fig. 3a). LAP adopts a wreath shape to encircle the mTGF-β1 dimer with ~3800 Å^2^ buried interface area (Fig. 1a–c). The straitjacket of LAP is linked to the lateral side of LRRC33 via covalent bonds. The Cys4 residues in the α1 helix from either LAP monomer form asymmetrical intermolecular disulfide bonds with Cys201 or Cys345 of LRRC33 (Fig. 1c, g, and h). The N-terminal region of LAP_A_ inserts into the hydrophobic core of LRRC33 from its convex side, facilitating the formation of the Cys4_A_-Cys201 bond (Fig. 1g). In contrast, the N-terminal region of LAP_B_ floats on the lateral surface of LRRC33 and further stabilizes their complex through van der Waals interactions (Fig. 1h). In addition to the covalent interaction, a non-covalent interface between LRRC33 and one mTGF-β1 molecule (mTGF-β1_B_) was also presented in our structure (Fig. 1i).

Compared to the previously reported structures^9,15,23,24^, L-TGF-β1 in its complex with LRRC33 displays three major local conformational characteristics. First, the α3 helix and preceding loop (Pro296-His317) of mTGF-β1_B_ flip over to accommodate the non-covalent interaction with LRRC33, similar to that observed in the L-TGF-β1/GARP complex (Fig. 1j and Supplementary Fig. 3a). Second, the N-terminal region including the α1 helix adopts a unique structure in our complex, suitable for disulfide bond formation with LRRC33 (Fig. 1k and Supplementary Fig. 3a). Third, the distal end of Arm domain, including the RGDLXX(L/I) motif and bowtie region, exhibits structural plastictiy in different L-TGF-β1 structures, which might provide enough mobility for integrin recognition (Fig. 1l and Supplementary Fig. 3a).

### Non-covalent interface essential for L-TGF-β1/LRRC33

The anchor proteins LTBP1, GARP, and LRRC33 are covalently linked to L-TGF-β1 via two disulfide bonds involving the conserved Cys4 residue of LAP^14,24,25^. However, non-covalent interactions between different anchor proteins and L-TGF-β1 are heterogeneous^25–28^. In our structure, mTGF-β1_B_ leans onto the lateral surface of LRRC33, resulting in a non-covalent interface of 535 Å^2^ (Fig. 1i). Trp301 of mTGF-β1_B_ inserts in a groove of LRRC33 between LRR fragments R7 and R8, forming hydrophobic interactions with Phe196 and Tyr217 of LRRC33, as well as hydrogen bond with the main-chain carboxyl group of Tyr217. At the same time, Tyr174 of LRRC33 is embedded into a hydrophobic pocket of mTGF-β1_B_ consisting of Pro298, Ile300, Leu313, and His317 (Fig. 1i and Supplementary Fig. 3b).

**Fig. 2:**
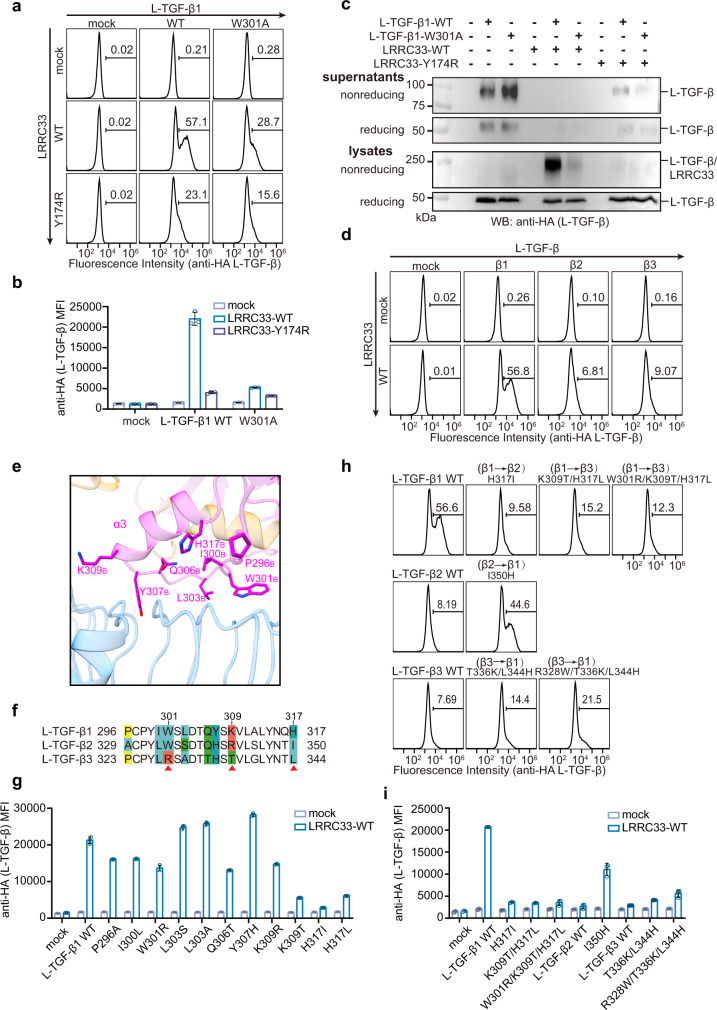
Non-covalent interaction is essential for the L-TGF-β1/LRRC33 assembly and determines L-TGF-β isoform specificity. **a** Surface L-TGF-β1 (anti-HA) expressions on transfected Expi293F cells were measured by flow cytometry. Number in each histogram indicates the percentage of L-TGF-β+ (anti-HA+) subset. **b** MFI (Mean Fluorescence Intensity) of L-TGF-β (anti-HA) on differently transfected Expi293F cells. All experiments were done in triplicate (*n* = 3 biologically independent experiments, mean ± s.d.). **c** Culture supernatants and total lysates of Expi293F cells transfected with indicated plasmids were subjected to non-reducing and reducing immunoblot analyses with anti-HA antibody (for L-TGF-β). The experiment was repeated three times independently with similar results. **d** Surface presentations of different L-TGF-β isoforms by LRRC33 were measured by flow cytometry. Number in each histogram indicates the percentage of L-TGF-β+ (anti-HA+) subset. **e** The non-covalent interface between mTGF-β1 and LRRC33. Side chains are shown for the mTGF-β1 residues that are in close distance with LRRC33. **f** Sequence alignment of the potential LRRC33-binding regions of the three L-TGF-β isoforms. Non-conserved residues are colored. **g** Surface presentation (MFI) of different L-TGF-β1 mutants by LRRC33. All experiments were done in triplicate (*n* = 3 biologically independent experiments, mean ± s.d.). **h**, **i** Cross mutations of the three key residues between different L-TGF-β isoforms switch their LRRC33-binding specificity. All experiments were done in triplicate (*n* = 3 biologically independent experiments, mean ± s.d.). Source data are provided as a Source Data file.

We next sought to determine the functional relevance of such extensive non-covalent interactions in the assembly of L-TGF-β1/LRRC33 complex by introducing mutations to the two key aromatic residues. Both the W301A mutation of L-TGF-β1 and the Y174R mutation of LRRC33 drastically decreased the L-TGF-β1/LRRC33 complex formation and the cell-surface presentation of L-TGF-β1 (Fig. 2a–c and Supplementary Fig. 4a). Y174R also impared LRRC33’s ability to withhold L-TGF-β1 from secretion (Fig. 2c). These results demonstrated that non-covalent interaction is critical in the assembly of L-TGF-β1 and LRRC33. Notably, unlike its homologous anchor protein GARP, LRRC33’s cell-surface expression depends on the coexpression of L-TGF-β1 (Supplementary Fig. 4b, c), suggesting that L-TGF-β1 acts as a molecular chaperone for LRRC33 during biosynthesis.

### LRRC33 specificity for L-TGF-β1

Anchor proteins, such as LTBP and GARP, have been reported with different specificity for the three TGF-β isoforms^8,13,29^. Our results showed that LRRC33 specifically presents L-TGF-β1, but not the β2 or β3 isoforms, to the plasma membrane (Fig. 2d and Supplementary Fig. 4d, e). In contrast, GARP efficiently presents all three isoforms (Supplementary Fig. 4f, g). The N-terminal Cys4 residue for covalent linkage to the anchor proteins is conserved in all three L-TGF-β isoforms, indicating the aforementioned non-covalent L-TGF-β1/LRRC33 interface is likely a key factor determining their specificity. We examined the regions surrounding the α3 helix (Pro296-His317) of mTGF-β1_B_ that predominantly mediates the non-covalent interactions with LRRC33 (Fig. 2e, f). Non-conserved residues within this region were systematically replaced with cognate residues of the β2 and β3 isoforms. Mutating His317 to the cognate residue of β2 (i.e., H317I) or β3 (i.e., H317L) severely impaired L-TGF-β1 presentation by LRRC33 (Fig. 2g and Supplementary Fig. 5a). Notably, such mutations did not affect L-TGF-β1 secretion nor cell surface presentation by GARP, ruling out any potential issue of protein expression or folding (Supplementary Fig. 5a, b). In addition, we found substituting Lys309 to the cognate residue of β3 (i.e., K309T) but not β2 (i.e., K309R) inhibited cell-surface presentation of L-TGF-β1 by LRRC33 (Fig. 2g and Supplementary Fig. 5a). Although the side-chain density of Lys309 in mTGF-β1_B_ is not directly visible in the L-TGF-β1/LRRC33 EM map, this residue likely participates in van der Waals interactions with Cys98 and Ser100 of LRRC33 based on the structural model (Supplementary Fig. 3c). Trp301, one of the two aforementioned key residues for L-TGF-β1/LRRC33 non-covalent interaction, is conserved in β1 and β2 but not β3. Mutating Trp301 to the cognate residue of β3 (i.e., W301R) also affected the L-TGF-β1 recognition (Fig. 2g and Supplementary Fig. 5a), although to a lesser extent compared to the W301A mutation (Fig. 2a). This is probably due to the partially negatively charged surface of LRRC33 that surrounds the Trp301 residue of mTGF-β1_B_ (Supplementary Fig. 3d).

To further validate the essential role of these three residues (Trp301, Lys309, and His317) for LRRC33 recognition, we introduced combined mutations in L-TGF-β1 to the cognate residues of β2 (H317I) or β3 (W301R/K309T/H317L). Both mutations dramatically abolished the assembly of the L-TGF-β1/LRRC33 complex (Fig. 2h, i and Supplementary Fig. 5c, d). In addition, reverse mutations back to the cognate residues of L-TGF-β1 were introduced to L-TGF-β2 or L-TGF-β3. Strikingly, a single mutation of Ile350 on L-TGF-β2 (i.e., I350H) was sufficient to restore its complex formation with LRRC33. Similarly, L-TGF-β3 with triple mutations of Arg328, Thr336, and Lys344 (i.e., R328W/T336K/L344H) could also be recognized and presented by LRRC33 (Fig. 2h, i and Supplementary Fig. 5c, d). However, this L-TGF-β3 variant did not fully recapitulate the binding of L-TGF-β1, indicating that other residues/regions may be at play.

### Specificity of the TGF-β1 activation by integrin α_V_β_8_

**Fig. 3:**
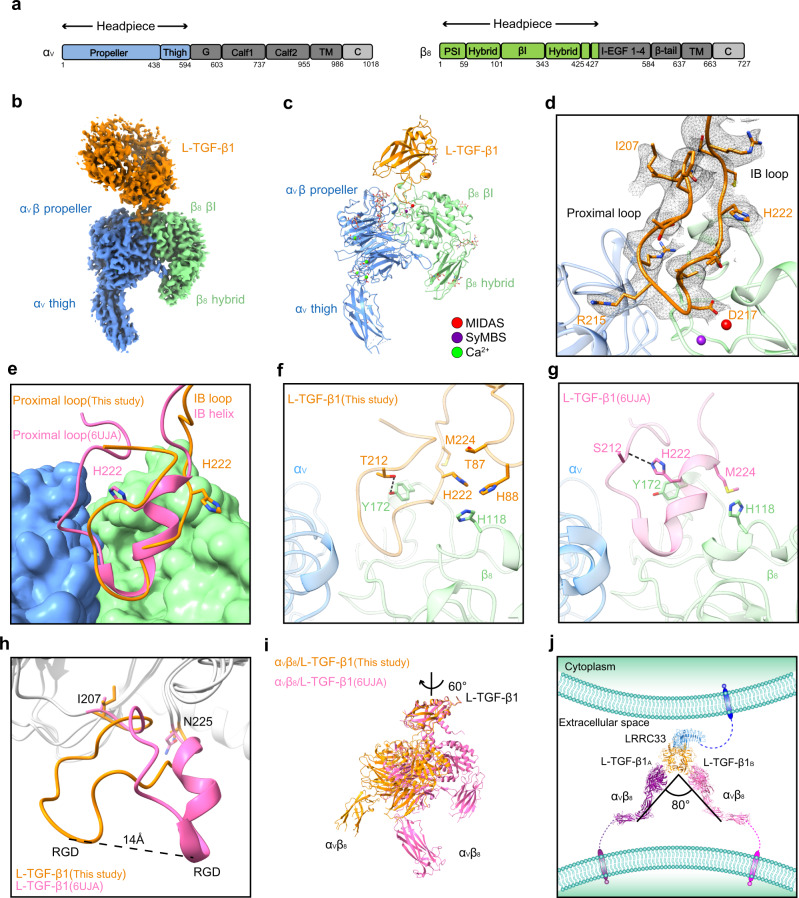
Cryo-EM structure of the α_V_β_8_/L-TGF-β1 complex. **a** Sequence schematic diagrams of integrin subunits α_V_ and β_8_. The headpiece regions of α_V_ and β_8_ used in our study are colored in blue and green, respectively. The other regions of integrin are colored in gray. **b** Cryo-EM map of the α_V_β_8_/L-TGF-β1 complex. The contour level is 0.384. The integrin subunits are colored as those in **a**. L-TGF-β1 is colored in orange. The cryo-EM density of LRRC33 is too weak to visualize. **c** Cartoon presentation of the α_V_β_8_/L-TGF-β1 structure built in the final model. Metal ions in the MIDAS (metal ion-dependent adhesion site) and SyMBS (syngeneic metal ion binding site) are colored in red and purple, respectively. Calcium ions are colored in green. **d** Ribbon diagram of the interface between α_V_β_8_ and L-TGF-β1_._ The side chains of the residues in the integrin-binding region of L-TGF-β1 are shown as sticks, and their cryo-EM densities (contour level of 0.384) are shown as black mesh. The proximal loop and integrin-binding (IB) loop are indicated. **e** Superposition of our α_V_β_8_/L-TGF-β1 structure with the reported one (PDB code: 6UJA) based on integrin α_V_β_8_. α_V_β_8_ is shown in surface presentation, and L-TGF-β1 is shown in ribbon. L-TGF-β1 in our structure is colored in orange, and that in the reported structure (PDB code: 6UJA) is colored in pink. The side chains of His222 in these two structures are shown as sticks. **f**, **g** Detailed comparison of the L-TGF-β1 integrin-binding regions between the two structures shown in **e**. The residues with distinct conformational changes are shown as sticks. Black dashed lines represent hydrogen bond interaction (<4 Å). **h** Superposition of the L-TGF-β1 Arm domain between our structure and the reported one (PDB code: 6UJA). The integrin-binding region exhibits a huge conformational difference. Two fixed residues, Ile207 and Asn225, are shown with side chains. **i** Superposition of our α_V_β_8_/L-TGF-β1 structure with the reported one (PDB code: 6UJA) based on L-TGF-β1. Integrin α_V_β_8_ rotates ~60 degrees along the vertical axis. **j** Assembled structural model for the 2:2:1 α_V_β_8_/L-TGF-β1/LRRC33 complex. The lower leg of integrin α_V_ (PDB code: 6AVU) is integrated into this model.

In contrast to integrin α_V_β_6_ which is predominantly expressed in epithelial cells, α_V_β_8_ is reported to be expressed in multiple immune cells that co-reside with L-TGF-β1 expressing myeloid lineage cells and is highly relevant in immune regulation^8,22^. Moreover, in the central nervous system (CNS), L-TGF-β1/LRRC33 activation in microglia depends on α_V_β_8_ but not α_V_β_6_^14^. To reveal the recognition and activation mechanisms of L-TGF-β1/LRRC33 by integrin α_V_β_8_, we next sought to pursue the cryo-EM structure of the α_V_β_8_/L-TGF-β1/LRRC33 ternary complex. Integrin α_V_β_8_ could bind L-TGF-β1 either in a ratio of 1:2 or 2:2^16^. We used a α_V_β_8_/L-TGF-β1/LRRC33 ternary complex of 2:2:1 stoichiometry for structural determination, in which integrin α_V_β_8_ contains only the headpiece involved in L-TGF-β1 binding (i.e., residues 1–594 of α_V_ subunit and residues 1–456 of β_8_ subunit) (Fig. 3a). Due to structural flexibility^19^ and pseudo-symmetric property of the ternary complex, only a medium-resolution structure (~5 Å) was achieved (Supplementary Fig. 6). To understand the interactions between integrin α_V_β_8_ and L-TGF-β1, we focused on one α_V_β_8_ headpiece and the L-TGF-β1 dimer within the EM map. Exquisite 3D classification showed that the EM densities of L-TGF-β1 and the relative position between α_V_β_8_ and L-TGF-β1 varied considerably between different subclasses, especially for the three classes with relatively higher resolution (Supplementary Fig. 7). This is in concert with the observed plasticity of L-TGF-β1 in the reported α_V_β_8_/L-TGF-β1 structure^19^. The best subclass of our 1:2 α_V_β_8_/L-TGF-β1 complex was finally refined to a resolution of 3.24 Å (Supplementary Figs. 6, 8 and Table 1), where the head region of α_V_β_8_ shows clear EM densities, including the β-propeller and proximal thigh domains of α_V_ subunit and the βI and hybrid domains of β_8_ subunit (Fig. 3b). In contrast to other integrins whose headpieces adopt an open conformation upon ligand binding^30^, the α_V_β_8_ headpiece remains closed in complex with L-TGF-β1^19,31^ (Fig. 3b, c). The absence of a metal ion in the conserved adjacent to metal ion-dependent adhesion site (ADMIDAS)^19,31^ is observed in our α_V_β_8_/L-TGF-β1 structure (Supplementary Fig. 9a).

The structure of α_V_β_8_ headpiece in our α_V_β_8_/L-TGF-β1 complex is almost identical to the reported ones either on its own or in complex with L-TGF-β1^19,31,32^ (Supplementary Fig. 9b). In terms of L-TGF-β1, the entire integrin-binding regions were well resolved in the density map, allowing us to build the corresponding structure model (Fig. 3d). In our structure, the RGD tri-peptide (^215−^RGD^−217^) of L-TGF-β1 inserts into the cleft between α_V_ β-propeller and β_8_ βI domains, similar to the reported α_V_β_8_/L-TGF-β1 and α_V_β_6_/L-TGF-β1 structures^15,19,31^. However, the adjacent proximal loop (^207−^INGFTTGR^−214^) and integrin-binding (IB) helix (^218−^LATIHGM^−224^) of L-TGF-β1 undergo tremendous conformational changes (Fig. 3e-g). Interestingly, rather than forming a continuous α-helix with ^218−^LATI^−221^, His222 of L-TGF-β1 flips outward and disrupts the secondary structure of the IB helix. Thus, we refer to the conventional IB helix as IB loop in our structure (Fig. 3e). The rotation of His222 breaks its hydrogen bond with the main-chain carboxyl group of Asn211, which helps to maintain the conventional proximal loop and IB helix structures in the 1:2 α_V_β_8_/L-TGF-β1 complex^19^. Instead, in our 2:2 α_V_β_8_/L-TGF-β1 complex, His222 is stabilized by Thr87 and His88 of L-TGF-β1, and His118 of integrin β_8_ through van der Waals interactions (Fig. 3f, g and Supplementary Fig. 9c). Moreover, Tyr172 of integrin β_8_ forms hydrogen bond with Thr212 to fix the proximal loop in the new position (Fig. 3f, g). This remodeling of proximal and IB loops of L-TGF-β1 leads to the movement of RGD motif by ~14 Å and the subsequent rotation of integrin α_V_β_8_ by ~60 degrees compared to the reported α_V_β_8_/L-TGF-β1 structure^19^ (Fig. 3h, i).

To obtain an intact structure model for the 2:2:1 α_V_β_8_/L-TGF-β1/LRRC33 complex, we fitted our α_V_β_8_/L-TGF-β1 and L-TGF-β1/LRRC33 structures into the medium-resolution EM map of the ternary complex (Supplementary Fig. 6). These two structures could be well aligned based on the common L-TGF-β1 component. A subsequent symmetric operation of the 1:2 α_V_β_8_/L-TGF-β1 model generated a 2:2 α_V_β_8_/L-TGF-β1 structure that nicely fits the EM map (Supplementary Fig. 9d), which helps confirm the structural model of the α_V_β_8_/L-TGF-β1/LRRC33 complex. This assembled model suggests that LRRC33 presents dimeric L-TGF-β1 on the surface of one cell, and two adjacent α_V_β_8_ molecules are able to bind L-TGF-β1 simultaneously with an included angle of ~80 degrees (Fig. 3j).

**Fig. 4:**
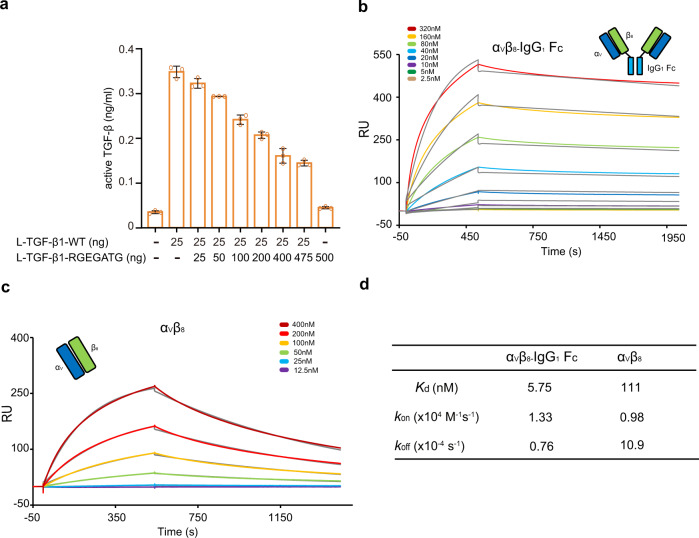
Functional significance of the 2:2 binding stoichiometry between integrin α_V_β_8_ and L-TGF-β1. **a** The measured TGF-β1 activity in (CAGA)_12_-Luciferase reporter cells when co-cultured with L-TGF-β1/GARP and integrin α_V_β_8_ expressing cells. The transfected amount of WT and integrin-binding defective (RGEGATG) L-TGF-β1 plasmids was indicated. All experiments were done in triplicate (*n* = 3 biologically independent experiments, mean ± s.d.). **b**, **c** The surface plasmon resonance (SPR) results for affinity measurements between L-TGF-β1 and two integrin α_V_β_8_ variants. Both the experimental (chromatic) and fitting (gray) curves are shown. α_V_β_8_-IgG_1_ Fc refers to a heterotetramer of α_V_β_8_ linked by IgG_1_ Fc fragment. As indicated, the concentrations of integrins used in the experiment were different in the two experiments. **d** Summarization of the dissociation constants (*K*_d_) and kinetic parameters (*k*_on_ and *k*_off_). Source data are provided as a Source Data file.

To explore the functional significance of the different binding stoichiometry between integrins and L-TGF-β1 (1:2, one integrin bound to one L-TGF-β1 dimer; and 2:2, two integrins simultaneously bound to one L-TGF-β1 dimer) for TGF-β1 activation, the following experiments were carried out. We made a mutant L-TGF-β1 construct with abolished integrin-binding ability by mutating the ^215−^RGDLATI^−221^ motif to RGEGATG (Supplementary Fig. 10a). Co-transfection of wild-type (WT) and mutant L-TGF-β1 constructs would yield three types of L-TGF-β1 dimer protein: WT L-TGF-β1 dimer (capable of binding two integrins), chimeric L-TGF-β1 dimer (capable of binding one integrin), and the mutant L-TGF-β1 dimer (no integrin binding). By comparing integrin-induced TGF-β1 activation of WT and chimeric L-TGF-β1 dimers, we could distinguish the activation efficiency of different integrin-binding ratios (2:2 or 1:2). Constant amount of WT L-TGF-β1 (25 ng) and GARP (500 ng) DNA were co-transfected with varied amount of mutant L-TGF-β1 DNA (0–475 ng) into Expi293F cells to display L-TGF-β1 onto the cell surface. Next, these cells were co-cultured with α_V_β_8_-expressing cells, and the TGF-β1 activity was measured using a transiently transfected (CAGA)_12_-Luciferase reporter cell line^33^. By gradually increasing the ratio of mutant to WT L-TGF-β1 DNA in co-transfection (0 ng:25 ng to 475 ng:25 ng), less WT L-TGF-β1 dimers, and more chimeric and mutant L-TGF-β1 dimers were displayed onto the cell surface (Supplementary Fig. 10b, c). Our results showed that, although the total amount of WT L-TGF-β1 subunit remained the same, L-TGF-β1 activation decreased gradually, suggesting that WT L-TGF-β1 dimers with two integrin-binding arms have higher activation efficiency than the chimeric dimer with only one integrin-binding arm (Fig. 4a and Supplementary Fig. 10b).

We speculated that the higher activation efficiency of the 2:2 binding mode is caused by the avidity effect. We integrated the Fc fragment of IgG_1_ (IgG_1_ Fc) to the C-terminal of integrin β_8_ subunit to tether two α_V_β_8_ integrins in a heterotetramer form which is able to bind the dimeric L-TGF-β1 at a 2:2 ratio (Fig. 4b). Surface plasmon resonance (SPR) results showed that the tetrameric integrin α_V_β_8_ has much higher affinity for L-TGF-β1 compared to WT α_V_β_8_ (*K*_d_ of 5.75 vs. 111 nM, respectively), demonstrating that the 2:2 binding mode indeed increases their binding avidity (Fig. 4b–d). Taken together, our results indicated that the 2:2 binding stoichiometry between integrin α_V_β_8_ and L-TGF-β1 is a biologically relevant state for TGF-β1 activation.

**Fig. 5:**
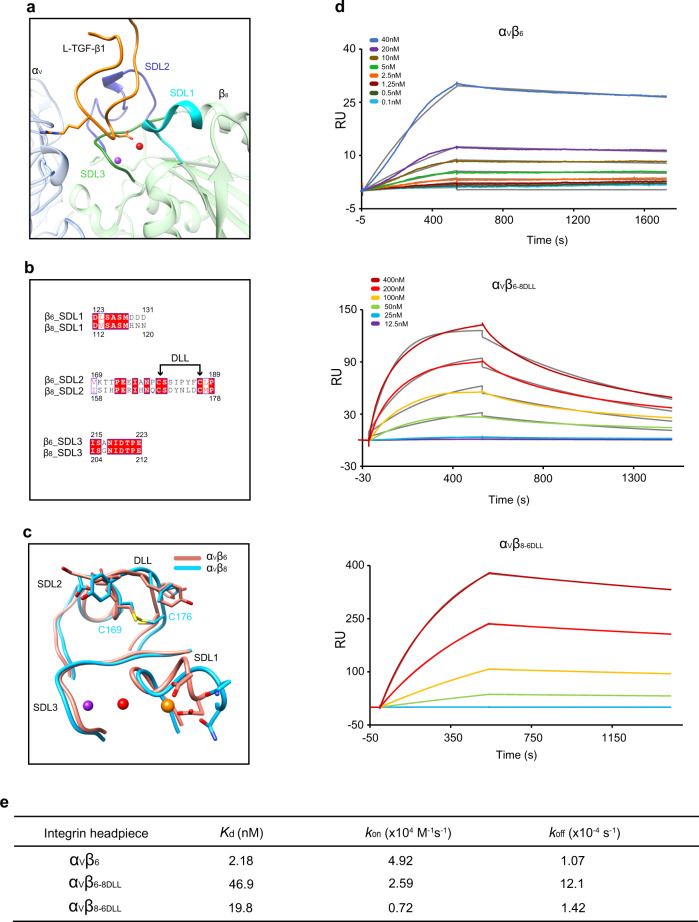
Structural and functional analyses of the disulfide-linked loop (DLL) regions of integrin subunits β_6_ and β_8_ for their contributions to L-TGF-β1 binding. **a** Ribbon diagram for the specificity-determining loops (SDLs) of α_V_β_8_ in the complex structure with L-TGF-β1. SDL1, cyan; SDL2, purple; SDL3, dark green. **b** Sequence alignment of the three SDLs between integrin subunits β_6_ and β_8_. DLL, disulfide-linked loop. **c** Structural comparison of SDLs in the structures of α_V_β_8_/L-TGF-β1 (blue) and α_V_β_6_/L-TGF-β1 (pink, PDB code: 5FFO). **d** The surface plasmon resonance (SPR) results for affinity measurements between L-TGF-β1 and different integrin variants. Both the experimental (chromatic) and fitting (gray) curves are shown. α_V_β_6-8DLL_, the DLL of β_6_ is substituted by that of β_8_; α_V_β_8-6DLL_, the DLL of β_8_ is substituted by that of β_6_. As indicated, the concentrations of α_V_β_6_ used in the experiment were different from those in the other two experiments. **e** Summarization of the dissociation constants (*K*_d_) and kinetic parameters (*k*_on_ and *k*_off_) for the binding between L-TGF-β1 and different integrin variants.

We then looked into the specificity of L-TGF-β1 binding to integrins α_V_β_6_ and α_V_β_8_. Both integrins can activate L-TGF-β1, but α_V_β_6_ has ~50-fold higher affinity for L-TGF-β1 than α_V_β_8_^16,17^. As the two integrins share the α_V_ subunit, the β subunits were examined in detail to elucidate their specific binding properties for L-TGF-β1. It has been shown that three specificity-determining loops (SDLs) contribute to the ligand recognition ability of integrin β subunits^34^. In the β_6_ subunit, SDL1 and SDL3 coordinate the conserved binding of ADMIDAS metal ions adjacent to the ligand-binding pocket (Fig. 5a). While the primary sequence and ternary structure of SDL3 are highly conserved between β_6_ and β_8_, the negatively charged Asp130 and Asp131 in the SDL1 of β_6_ are substituted by Asn119 and Asn120 in β_8_ (Fig. 5b), which causes the lack of ADMIDAS metal ions in β_8_ (Fig. 5c and Supplementary Fig. 9a). However, simultaneously mutating Asn119 and Asn120 of β_8_ to Asp only showed a modest effect on the L-TGF-β1 affinity of α_V_β_8_^31^. Therefore, SDL1 and SDL3 of β subunits are unlikely the primary determinant for the different L-TGF-β1 affinity.

On the other hand, we noted that 5 out of 8 residues of the disulfide-linked loop (DLL) within SDL2 are different between β_6_ and β_8_ (Fig. 5b). Moreover, although the main chains of DLLs in β_6_ and β_8_ are similar upon L-TGF-β1 binding, the side chains of DLLs adopt significantly different conformations (Fig. 5c). We thus swapped the DLL of β_8_ to β_6_ and prepared the mutant integrin headpiece protein (i.e., α_V_β_6-8DLL_), and vice versa (i.e., α_V_β_8-6DLL_). The L-TGF-β1 affinity of such mutant integrin proteins was then measured by SPR. The WT α_V_β_6_ bound L-TGF-β1 with a *K*_d_ value of 2.18 nM, while that of α_V_β_8_ was 111 nM, in accordance with the higher binding affinity of α_V_β_6_ to L-TGF-β1. In contrast, the L-TGF-β1 affinities of mutant α_V_β_6-8DLL_ (*K*_d_ = 46.9 nM) and α_V_β_8-6DLL_ (*K*_d_ = 19.8 nM) were shifted towards that of α_V_β_8_ and α_V_β_6_, respectively. Interestingly, the *k*_off_ values were more affected than the *k*_on_ values (Figs. 4c, d, and 5d, e). It was reported that the headpiece opening of integrin α_V_β_6_ contributes to its higher affinity for L-TGF-β1 than integrin α_V_β_8_ that remains in the closed conformation upon L-TGF-β1 binding. We observed that α_V_β_6-8DLL_ and α_V_β_8-6DLL_ maintain their intrinsic open and closed conformations in the complexes with L-TGF-β1, respectively (Supplementary Fig. 11a, b), ruling out the possibility that the DLL swapping between β_6_ and β_8_ would change the ligand affinity via the effect on their inherent conformational adjustment. Together, the results identified the DLL within SDL2 of β_6_ and β_8_ as the key determinant for their different affinities for L-TGF-β1.

## Discussion

TGF-β signaling is broadly involved in the development, homeostasis, and diseases^1,7,22,35–37^. Extensive studies have demonstrated that TGF-β1, TGF-β2, and TGF-β3 exert intrinsically distinct biological activities^38–40^, although the three isoforms initiate the same downstream signaling events via TGF-β receptors. Moreover, even the same isoform could carry out drastically different functions in different physiological contexts. Consequently, therapeutic applications against the TGF-β pathway has been greatly hindered due to severe adverse side effects caused by blocking TGF-β’s function systematically^35^. Therefore, uncovering the mechanism designating the specificity of TGF-β signal is central to the biological understanding of the TGF-β pathway.

Recent studies on the activation and extracellular deposition of the latent TGF-β precursor provide an alternative perspective for the mechanisms underlying the functional specificity of TGF-β^14–17,19,23,24^. TGF-β is synthesized as a latent form (L-TGF-β). It becomes increasingly evident that specific distribution of L-TGF-β in the extracellular microenvironment and the release of mature TGF-β from the latent form are the two critical checkpoints for the functional divergence of the three TGF-β isoforms. For instance, the anchor proteins LTBP-1 and -3 can present all the three TGF-β isoforms, but LTBP-4 only binds TGF-β1^41^. Therefore, different expression patterns of such LTBPs would help determine the signaling heterogeneity of TGF-β isoforms in various biological contexts^42^. Of importance, LRRC33 is identified as a L-TGF-β anchor protein on the surface of myeloid lineage cells. LRRC33-deficient mice feature impared TGF-β1 signaling, resulting in aberrant activation of microglia and multiple neurological disorders in the CNS^14,43^. In addition, different myeloid cells in the cancer microenvironment, e.g., myeloid-derived suppressor cells, tumor-associated macrophages, and dendritic cells, have been reported to express high levels of LRRC33 and thus to be the major sources of TGF-β1^44^, whose immunosuppressive function contributes to tumor progression and leads to failure in anti-PD-1/PD-L1 therapies in certain types of cancer^45^.

**Fig. 6:**
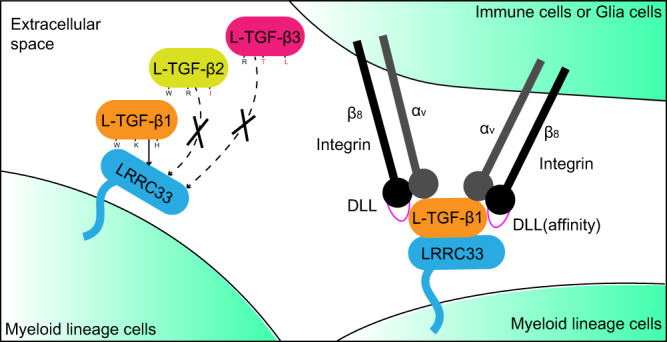
Schemetic model for the specific TGF-β1 signal of the myeloid lineage cells designated by LRRC33 and integrin α_V_β_8_. The three key residues that determine the specific cell-surface L-TGF-β1 presentation by LRRC33 are labeled. The cognate residues of L-TGF-β2 and L-TGF-β3 are also indicated with the major discriminative ones colored in red.

Notably, LRRC33-deficiency only exhibits TGF-β1-, but not β2- or β3-, related deficits. The mechanism underlying the specific L-TGF-β1 presentation by LRRC33 and their functional correlation had not been defined until this work. By solving the L-TGF-β1/LRRC33 complex structure, we showed that LRRC33 only presents L-TGF-β1 on the cell surface, and revealed that non-covalent interactions between L-TGF-β1 and LRRC33 are the determinant for their specific pairing. Replacing three key residues on L-TGF-β2 or -β3 with the cognate ones of L-TGF-β1 that mediate such non-covalent interactions was sufficient to enable LRRC33 presentation of these two isoforms (Figs. 2h, i and 6). Moreover, our findings here suggest that cell surface expression of LRRC33 directly correlates with L-TGF-β1, ie. it cannot be expressed by itself (Supplementary Fig. 4b, c). These results together indicate that LRRC33 will be a promising drug target for specifically blocking the myeloid-derived TGF-β1 signal in therapeutic applications, such as cancer immunotherapy, just like the GARP-targeted TGF-β1 inhibition for T_reg_ cells^46^. The key non-covalent interaction site we characterized in this work also provides the molecular basis for small molecules design or antibodies development to intervene in L-TGF-β1 presentation and its subsequent functions.

Unlike TGF-β-expressing non-immune cells, myeloid lineage cells can migrate within tissues and encounter different microenvironments. Therefore, in addition to the specific presentation of the L-TGF-β1 isoform by LRRC33, mTGF-β1 release from the latent complex serves as another essential layer of regulation. As aforementioned, activation by α_V_β_8_ is more relevant in the context of myeloid lineage cells. The underlying mechanism, however, is still obscure. Both integrins α_V_β_6_ and α_V_β_8_ binds to the RGDLXXL/I motif of L-TGF-β1, yet it remains unclear how α_V_β_6_ and α_V_β_8_ exert significantly different ligand affinities. Here we demonstrated it is SDL2 of integrin β subunits that designates their L-TGF-β1 binding affinities mainly by determining the off-rate (Figs. 4c, d, 5, and 6). Swapping the DLL region of SDL2 between α_V_β_6_ and α_V_β_8_ could interchange their affinities for L-TGF-β1. Accordingly, the DLL region of integrins could represent an important entry point for antibodies-mediated targeting of the TGF-β1 signal.

Another important question in integrin-mediated L-TGF-β1 activation is the stoichiometry and orientation of their binding. Our 2:2 α_V_β_8_/L-TGF-β1 complex structure shows a markedly different binding mode compared to the previously reported 1:2 integrin/L-TGF-β1 complexes^15,19^. When two α_V_β_8_ are bound simultaneously, each integrin rotates 60 degrees along the vertical axis, and forms an 80 degrees angle (Fig. 3i, j). This is in great contrast to the modeled two α_V_β_8_ binding pattern based on the 1:2 α_V_β_8_/L-TGF-β1 complex structure^19^, which shows a much larger separation of the two integrins (150 degrees) (Supplementary Fig. 11c). Moreover, the α_V_β_8_/L-TGF-β1 interface is also different in our 2:2 complex structure. The LATI motif forms a loop (IB loop) instead of the signature α-helical conformation^17,19,31^, and the buried interface is 19% smaller compared to that of the 1:2 complex (740 Å^2^ vs. 910 Å^2^)^19^. The huge conformational change from the 1:2 to 2:2 α_V_β_8_/L-TGF-β1 complexes is possibly due to the spatial restrictions of the two integrin-binding motifs residing on the adjacent disulfide-linked bowtie tail loops from each L-TGF-β1 monomer. Once two integrins bind L-TGF-β1 concurrently, their local interfaces need to be rearranged to better accommodate each other. Alternatively, it is also possible that the different structures represent two intermediate states in the process of L-TGF-β1 activation by integrins. Overall, our structural and functional analyses suggest that a 2:2 binding stoichiometry is physiologically relevant. Given the narrow separation and the flexibility of integrin α_V_β_8_ legs, two α_V_β_8_ integrins on one cell are able to simultaneously bind to one L-TGF-β1 presented by LRRC33 on a neighboring cell (Figs. 3j and 6). It is possible that the traction force between two adjacent cells provides the mechanical force for LAP dissociation, during which process high avidity caused by simultaneous binding of two α_V_β_8_ to one L-TGF-β1 guarantees the efficient force transmission. In this way, mTGF-β1 could be released and activates distant cells, not just limited to the L-TGF-β1 presenting cells^19^. Notably, the structural model here was concluded from the truncated headpiece of integrins. As for the full-length α_V_β_8_, its interaction mode with L-TGF-β1 might look different.

In sum, this work identified the mechanism underlying the exquisite specificity of TGF-β1 presentation and activation, and provided molecular basis for the design of therapeutic agents specifically targeting myeloid-derived TGF-β1 signal.

## Methods

### Cell cultures

Sf9 cells (ATCC CRL-1711) were cultured in Sf-900 II SFM medium (Gibco) at 27 °C. HEK293S GnTI^−^ cells (ATCC CRL-3022) were cultured in Yocon HEK293 medium (Yocon Biotechnology) supplemented with 1% fetal bovine serum (FBS) (Vistech) and 100 μg/ml penicillin/streptomycin (Gibco) at 37 °C with 5% CO_2_. Expi293F cells (Gibco A14527) were cultured in OPM-293 CD05 medium (OPM, 81075-001) in suspension or FreeStyle 293 medium (Gibco) adherently at 37°C with 5% CO_2_.

### Protein expression and purification

For L-TGF-β1 and LRRC33, the DNA sequences encoding the full-length L-TGF-β1 (residues 1–361 excluding signal peptide) and LRRC33 ectodomain (residues 1–631 excluding signal peptide) were cloned into the pEG BacMam expression vector^47^. 6 × His-tag was attached either to the N-terminus of L-TGF-β1 or the C-terminus of LRRC33. As for integrins, the DNA sequences encoding the headpieces of subunits α_V_ (residues 1–594 excluding signal peptide), β_6_ (residues 1–474 excluding signal peptide), and β_8_ (residues 1–456 excluding signal peptide) were cloned into the same pEG BacMam vector, and GFP tag was attached to the C-terminus of β subunits. β_6-8DLL_ and β_8-6DLL_ were generated using Gibson assembly protocol. All the plasmids were transformed into DH10Bac *Escherichia coli* competent cells for bacmid generation, and then the recombinant baculoviruses were generated using sf9 insect cells. For the expression of L-TGF-β1 alone, 10% His-tagged passage 3 (P3) virus was added to HEK293S GnTI^−^ cells at a density of 3 × 10^6^ cells/ml. For the expression of the L-TGF-β1/LRRC33 complex and different integrins (α_V_β_6_, α_V_β_8_, α_V_β_6-8DLL_, and α_V_β_8-6DLL_), equal amount of P3 viruses of untagged L-TGF-β1 (5%) and His-tagged LRRC33 (5%), or α_V_ (5%) and the corresponding β subunits (5%), were added together into HEK293S GnTI^−^ cells. 10 mM sodium butyrate was supplemented after 8 h to induce protein expression. The conditioned media were used for protein purification after 3 days’ culture.

For protein purification, the conditioned media were first centrifuged at 1700 × *g* for 20 min, and then the supernatants were concentrated and exchanged into Buffer A (20 mM Tris pH 8.0 and 500 mM NaCl) using a Hydrosart Ultrafilter system (Sartorius). His-tagged L-TGF-β1 or L-TGF-β1/LRRC33 complex were mixed with Ni-NTA beads (Smart-Lifesciences) at 4 °C for 2 h. Next, the beads were washed in turn with Buffer A and B (20 mM Tris pH 8.0, 500 mM NaCl, and 20 mM imidazole) for 20 column volumes (CVs), respectively. Then the proteins were eluted in buffer C (20 mM Tris pH 8.0, 500 mM NaCl, and 200 mM imidazole), followed by cleavage of the his-tag via incubation with PreScission protease at 4 °C overnight. Instead, GFP-tagged integrins were mixed with anti-GFP nanobody (GFPnb)-coupled cyanogen bromide-activated Sepharose beads (GE Healthcare) at 4 °C for 2 h. After washing with Buffer A for 20 CVs, the beads were incubated with PreScission protease at 4 °C overnight to release the target proteins. Finally, all the proteins were further purified by size exclusion chromatography (SEC) using a Superose 6 Increase 10/300 GL column (GE Healthcare) equilibrated with Buffer D (20 mM HEPES pH 7.5 and 150 mM NaCl). To prepare the 2:2:1 α_V_β_8_/L-TGF-β1/LRRC33 ternary complex, purified integrin α_V_β_8_ and L-TGF-β1/LRRC33 were mixed on ice for 30 min in a molar ratio of 2.2:1 with the addition of 1 mM MnCl_2_ and 0.2 mM CaCl_2_. Mn^2+^ was used here instead of Mg^2+^ to strengthen the binding of integrin to L-TGF-β1^16^. The extra integrin was removed by another round of SEC in Buffer E (20 mM HEPES pH 7.5, 150 mM NaCl, 1 mM MnCl_2_, and 0.2 mM CaCl_2_). To prepare the α_V_β_8-6DLL_/L-TGF-β1 and α_V_β_6-8DLL_/L-TGF-β1 binary complexes, purified integrins and L-TGF-β1 were mixed on ice for 30 min in a molar ratio of 2.2:1 with the addition of 1 mM MgCl_2_ and 1 mM CaCl_2_, followed by SEC in buffer F (20 mM HEPES pH 7.5, 150 mM NaCl, 1 mM MgCl_2_, and 1 mM CaCl_2_).

### Cryo-EM sample preparation and data collection

The protein samples of L-TGF-β1/LRRC33 and α_V_β_8_/L-TGF-β1/LRRC33 from the peak fractions of SEC were concentrated to 3–4 mg/ml. 3 μl samples were deposited onto glow-discharged holey-carbon gold grids (Quantifoil), and then blotted, and flash-frozen in liquid ethane using Vitrobot Mark IV (FEI). The blot time was 2 s, humidity was 100%, and temperature was 12 °C.

The grids were first screened using a 200 kV Talos Arctica electron microscope (FEI). The ones in good quality were saved and used for data collection on a 300 kV Titan Krios electron microscope (FEI) equipped with a Gatan imaging filter (20 eV slit). Data acquisition was performed automatically using SerialEM software^48^. All the micrographs were recorded by a K2 Summit direct electron detector (Gatan) in super-resolution mode with a physical pixel size of 1.055 Å. The dose rate was 10 electrons/pixel/second and the total exposure time was 8 s. Each dataset was collected in a single session with a nominal defocus range of 0.8–1.2 μm. Three datasets were collected for the L-TGF-β1/LRRC33 sample, which includes 2718, 3031, and 1436 micrographs, respectively. One dataset with 6711 micrographs was collected for the α_V_β_8_/L-TGF-β1/LRRC33 ternary complex.

### Cryo-EM data processing

The micrographs were motion corrected using MotionCor2^49^, contrast transfer function (CTF) was estimated using Gctf^50^, and particles were auto-picked using Gautomatch (http://www.mrc-lmb.cam.ac.uk/kzhang). The following classifications and refinements were mainly performed in RELION 3.1^51^. Non-uniform (NU) and local refinements of cryoSPARC^52^ were carried out for the final refinement.

For the three datasets of L-TGF-β1/LRRC33, all the extracted particles were combined for data processing. Good classes of 2D classification were input to 3D classification. Then the best classes were combined for 3D auto-refine and produced a 5.10-Å map. A second round of 3D classification without alignment further removed heterogeneous particles and improved the resolution to 4.82 Å. Next, except for a relatively low-resolution class, 5 classes (90.6% particles) from the first round of 3D classification were applied to another round of 3D classification with the 4.82-Å map as reference. From here, the two best classes were selected and refined to 4.74-Å resolution. The two sets of particle stacks that produced the 4.82-Å and 4.74-Å map were then combined and cleaned up by an extra round of 3D classification. The best classes were used for the final refinement. Bayesian polishing and CTF refinement were performed right after to further improve the resolution to 4.66 Å. Then the particles were refined using NU refinement in cryoSPARC, which boosted the resolution to 4.11 Å. Finally, the map was refined to 4.01-Å resolution by implementing a local refinement in cryoSPARC. The post-processed map was generated by DeepEMhancer^53^.

For the α_V_β_8_/L-TGF-β1/LRRC33 dataset, the 2D classes showed a clear view of 2:2:1 complex. However, 3D classification and auto-refine only generated a 5.19-Å map. To improve the resolution, a mask only containing the 1:2 α_V_β_8_/L-TGF-β1 complex was applied for the following processing. Two rounds of sequential 3D classification and auto-refine produced a 3.85-Å map. Like the previous dataset, Bayesian polishing, CTF refinement, and the following NU and local refinements finally boosted the resolution to 3.24 Å. Additionally, another round of exquisite 3D classification was performed to verify the plasticity of this complex in our dataset. All the resolutions reported here were calculated using the 0.143 cutoff criterion.

### Model building and refinement

The reported structures of α_V_β_8_ (PDB code: 6UJA) and L-TGF-β1 (PDB code: 6GFF) were directly used for our model building, while the initial model of LRRC33 was generated using SWISS-MODEL^54^. All these models were roughly fitted into the cryo-EM maps of L-TGF-β1/LRRC33 and α_V_β_8_/L-TGF-β1 using Chimera^55^, then they were manually adjusted using Coot^56^. Refinement of the final structure in real space was done by PHENIX^57^. The geometries of the model were validated using MolProbity^58^. The Fourier shell correlation (FSC) curves were calculated between the refined model and masked full map. Local resolution was estimated in cyroSPARC. All the figures were prepared using Chimera and ChimeraX^59^.

### Negative staining EM sample preparation, data collection, and processing

The protein samples of α_V_β_6-8DLL_/L-TGF-β1 and α_V_β_8-6DLL_/L-TGF-β1 from the peak fractions of SEC were diluted to 0.01 mg/ml. 5 μl samples were deposited onto glow-discharged carbon-coated copper grids (EMCN) for 1 min. The grids were then blotted by filter paper and stained with 2% uranyl acetate. After staining, the grids were dried in air and examined under JEOL JEM-F200 electron microscopy operated at 200 kV. The grids in good quality were used for data collection with a Gatan Oneview camera (Gatan). One dataset with 22 micrographs was collected for α_V_β_6-8DLL_/L-TGF-β1, and another one with 70 micrographs was collected for α_V_β_8-6DLL_/L-TGF-β1. 11,023 particles from the α_V_β_6-8DLL_/L-TGF-β1 dataset and 34,851 particles from the α_V_β_8-6DLL_/L-TGF-β1 dataset were used for 2D classification, respectively.

### Surface plasmon resonance (SPR) analysis

The SPR assay was conducted at 25 °C using a Biacore T200 instrument with its inbuild control software (GE Healthcare). WT (C4S/R249A) or mutated (C4S/R249A + RGEGATG) L-TGF-β1 was immobilized on a CM5 chip through amine coupling. Soluble integrin headpieces of α_V_β_6_, α_V_β_8_, α_V_β_6-8DLL_, α_V_β_8-6DLL_, and α_V_β_8_-IgG_1_ Fc were prepared in a series of indicated concentrations and injected in turn at a flow rate of 20 μl/min in HBS buffer (20 mM HEPES pH 7.5, 150 mM NaCl, 1 mM MgCl_2_, and 1 mM CaCl_2_). The surface was regenerated using 10 mM glycine pH 1.5 by a 30-s pulse (50 μl/min) at the end of each cycle to restore the resonance units to the baseline. Kinetics analyses were performed using Biacore evaluation software. The experimental data were fitted to a 1:1 Langmuir binding model to generate the kinetics parameters.

### L-TGF-β-anchor protein complex formation and surface presentation analysis

Genes encoding N-terminal Flag-tagged GARP and LRRC33 (WT and Y174R) were cloned into pD2529 mammalian expression vector (ATUM). To boost the cell-surface expression of LRRC33, its C-terminal transmembrane domain was substituted with that of GARP^14^. Genes encoding L-TGF-β1, -β2, and -β3 (WT and mutants) were cloned into pcDNA3.4 mammalian expression vector (Invitrogen). The flexible furin cleavage site between LAP and the growth factor of L-TGF-β was replaced with HA-tag to prevent dissociation of L-TGF-β and to avoid interfering with complexes formation (N-terminus is involved in complex formation with LRRC33 and GARP, thus N-terminal tagging is not optimal). Mutants were generated by two-step PCR using PrimeSTAR® HS DNA Polymerase (Takara, R044A) and cloned into Top10 *Escherichia coli* competent cells (Tsingke, TSC-C12) using ClonExpress MultiS One Step Cloning Kit (Vazyme, C113-02). All mutations were validated by DNA sequencing. Transiently transfected Expi293F cells were stained with iFluor 488 conjugated HA antibody (1 μg/ml, Genscript, Cat A01806, Clone 5E11D8) and iFluor 647 conjugated Flag antibody (1 μg/ml, Genscript, Cat A01811-100, Clone 5A8E5), and subjected to flow cytometry using a CytoFLEX cytometer (Beckman Coulter). The results were analyzed using FlowJo V10. Supernatants and total lysates of the transfected cells, were subjected to non-reducing and reducing SDS-PAGE for immunoblotting with anti-HA primary antibody (1:2000, Biolegend, Cat 901501, Clone 16B12) and anti-Mouse IgG (whole molecule) HRP secondary antibody (1:30000, Sigma, Cat A9044) to detect L-TGF-β and its complex with LRRC33 or GARP.

### L-TGF-β activation assay

Genes encoding L-TGF-β1 WT with an N-terminal Flag tag and a L-TGF-β1 variant (RGDLATI->RGEGATG) with an N terminal HA tag were cloned into pcDNA3.4 mammalian expression vector. Expi293F cells were transfected with constant amount of GARP (500 ng) and L-TGF-β1 WT plasmids (25 ng), but gradually increased amount of L-TGF-β1 variant plasmid (0–475 ng). Transiently transfected Expi293F cells were stained with iFluor 488 conjugated HA and iFluor 647 conjugated Flag antibodies, and analyzed using flow cytometry to determine the cell surface expression of L-TGF-β1 WT and variant.

5000 transfected cells described above were co-cultured with 5000 integrin α_V_β_8_ transfected Expi293F cells and 20,000 Expi293F cells transfected with a TGF-β-SMAD3 responsive (CAGA)_12_-Luciferase reporter construct. After 24 h of incubation, cells were lysed for 30 min on ice using 50 μl per well 1× Passive Lysis Buffer (Promega, E1941). Then the supernatants were transferred into a 96-well solid white flat microplate (Corning, 3917). 100 μl luciferase substrate was added per well according to the manufacturer’s recommendations (Promega, E1501) and the chemiluminescence was measured using a Synergy H1 microplate Reader (Bio Tek). A standard curve was calculated with serial diluted purified mTGF-β1 protein (Genscript).

## Supplementary information

Supplementary Information

## Data Availability

The cryo-EM density maps of L-TGF-β1/LRRC33, integrin α_V_β_8_/L-TGF-β1 (1:2), and α_V_β_8_/L-TGF-β1/LRRC33 (2:2:1) have been deposited in the Electron Microscopy Data Bank under the accession codes EMD-33571, EMD-33572, and EMD-33573. The atomic coordinates of L-TGF-β1/LRRC33 and α_V_β_8_/L-TGF-β1 have been deposited in the Protein Data Bank under accession codes 7Y1R and 7Y1T. Source data are provided with this paper.

## Source data

Source Data
