# Supplementary material for: Specificity of TGF-β1 signal designated by LRRC33 and integrin αVβ8
Source: Nat Commun. 2022 Aug 25;13:4988. doi: 10.1038/s41467-022-32655-9 (PMC9411592; doi:10.1038/s41467-022-32655-9)
Supplement: Supplementary file 1 — Supplementary Information [file 41467_2022_32655_MOESM1_ESM.pdf]

Supplementary Information for

Specificity of TGF-β1 Signal Designated by LRRC33 and Integrin $\alpha_V\beta_8$

Zelin Duan[1#], Xuezhen Lin[2#], Lixia Wang[2#], Qiuxin Zhen[1], Yuefeng Jiang[1], Chuxin Chen[1], Jing Yang[1], Chia-Hsueh Lee[3], Yan Qin[4], Ying Li[2], Bo Zhao[2*], Jianchuan Wang[5*], Zhe Zhang[1*]

[1]State Key Laboratory of Membrane Biology, Center for Life Sciences, School of Life Sciences, Peking University, Beijing 100871, China

[2]Molecular Cancer Research Center, School of Medicine, Shenzhen Campus of Sun Yat-sen University, No. 66, Gongchang Road, Guangming District, Shenzhen, Guangdong 518107, China

[3]Department of Structural Biology, St. Jude Children's Research Hospital, Memphis, TN 38105, United States

[4]Fuhong Therapeutics, 99 Hayden Ave d100, Lexington, MA 02421, United States

[5]Center for Translational Research, Shenzhen Bay Laboratory, Shenzhen, Guangdong 518007, China

[#]These authors contributed equally to this work

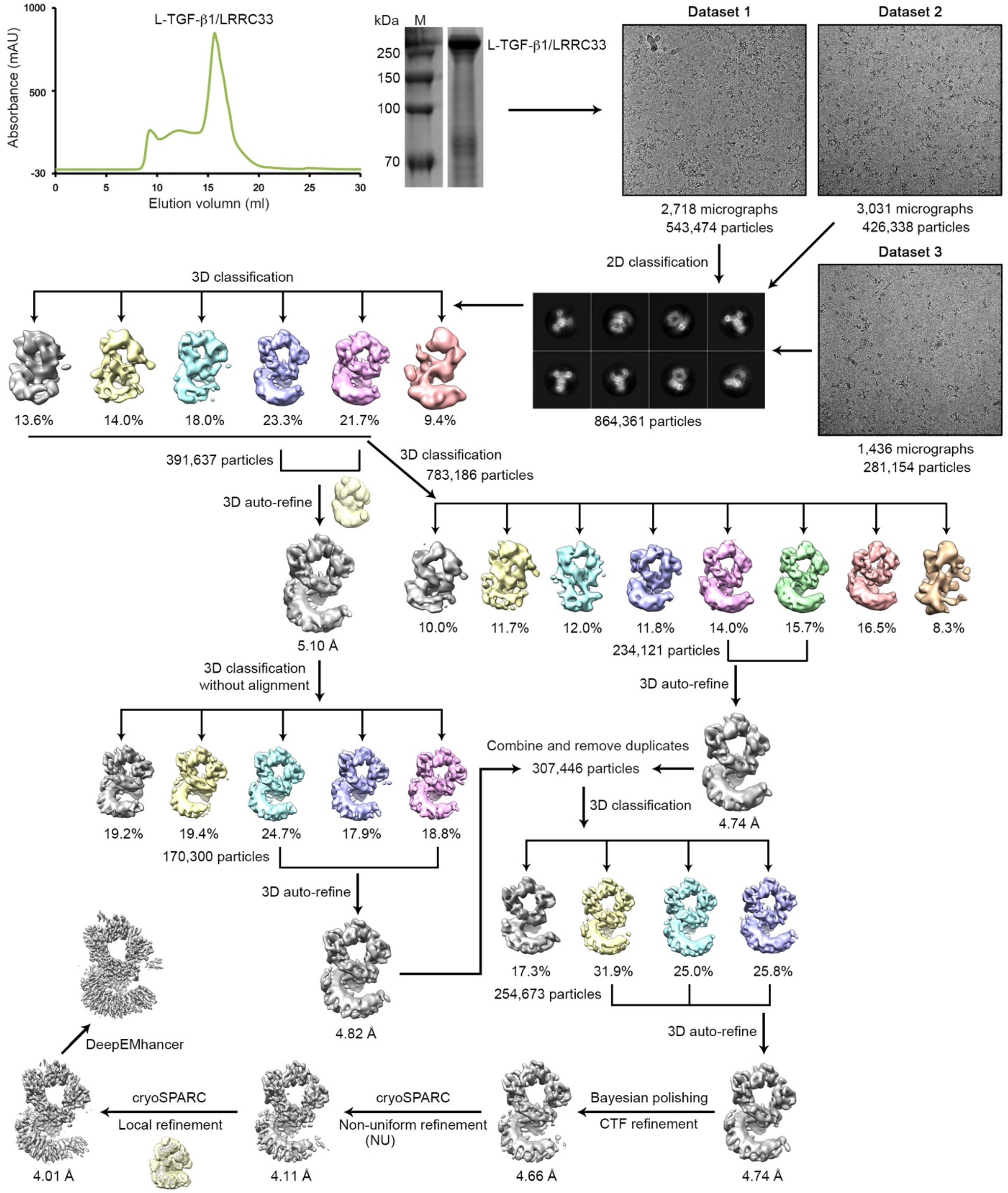

Supplementary Fig. 1 Cryo-EM analysis of the L-TGF-β1/LRRC33 complex.

Work flow for the purification and cryo-EM data processing of the L-TGF-β1/LRRC33 complex. The size exclusion chromatography (SEC) experiment was repeated three times independently with similar results. Source data are provided as a Source Data file.

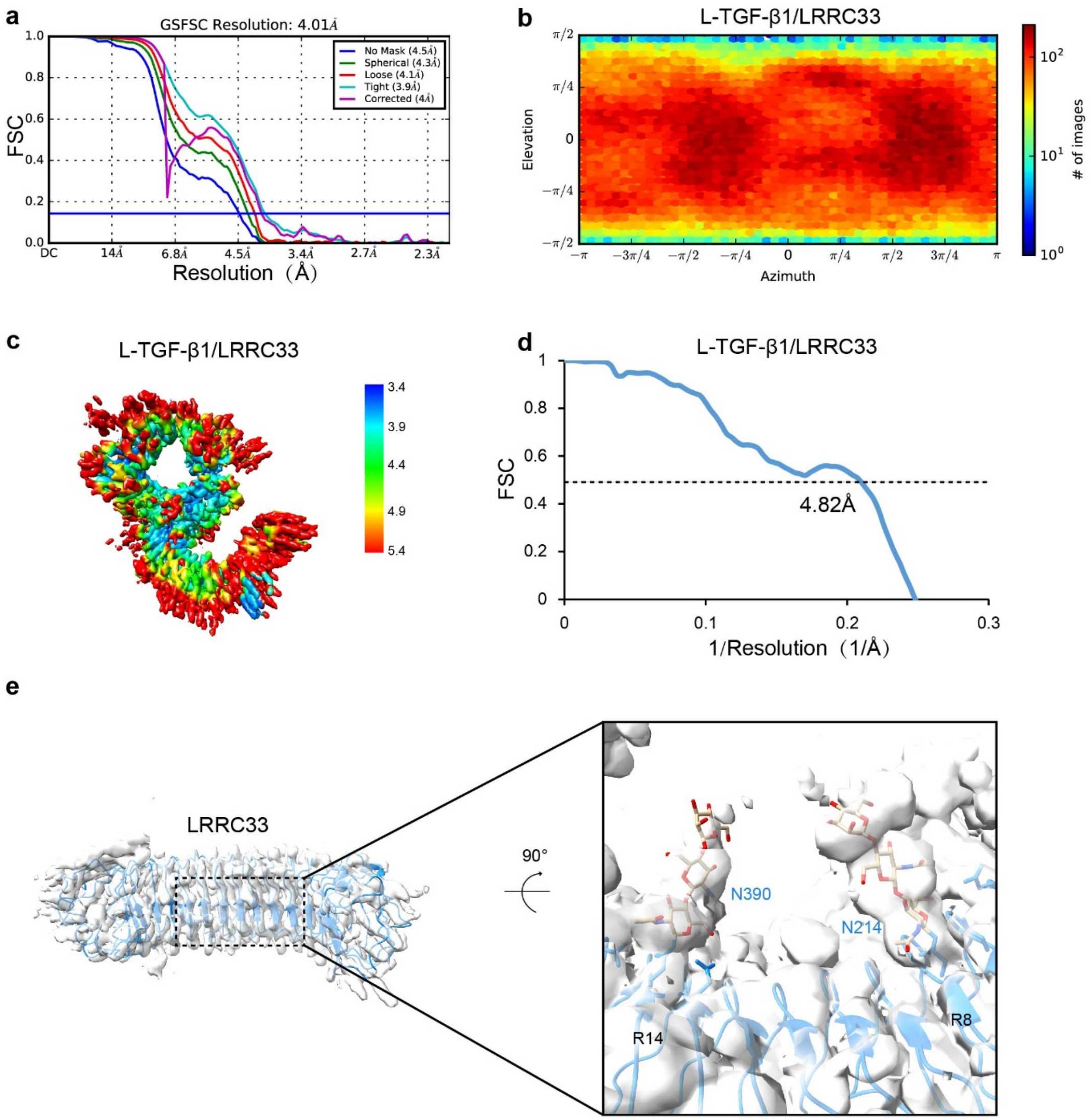

Supplementary Fig. 2 Assessment of the cryo-EM data and structural model for the L-TGF-β1/LRRC33 complex.

a Fourier Shell Correlation (FSC) curves between reconstructions of two half-datasets. **b** Angular distributions of all the particles used for the reconstruction. **c** Local resolution estimation for the cryo-EM map. The contour level is 0.294. **d** FSC curve between the refined structure and the cryo-EM map. **e** Cryo-EM densities of LRRC33. The contour level is 0.251. The linked N-glycans to Asn214 and Asn390 are indicated.

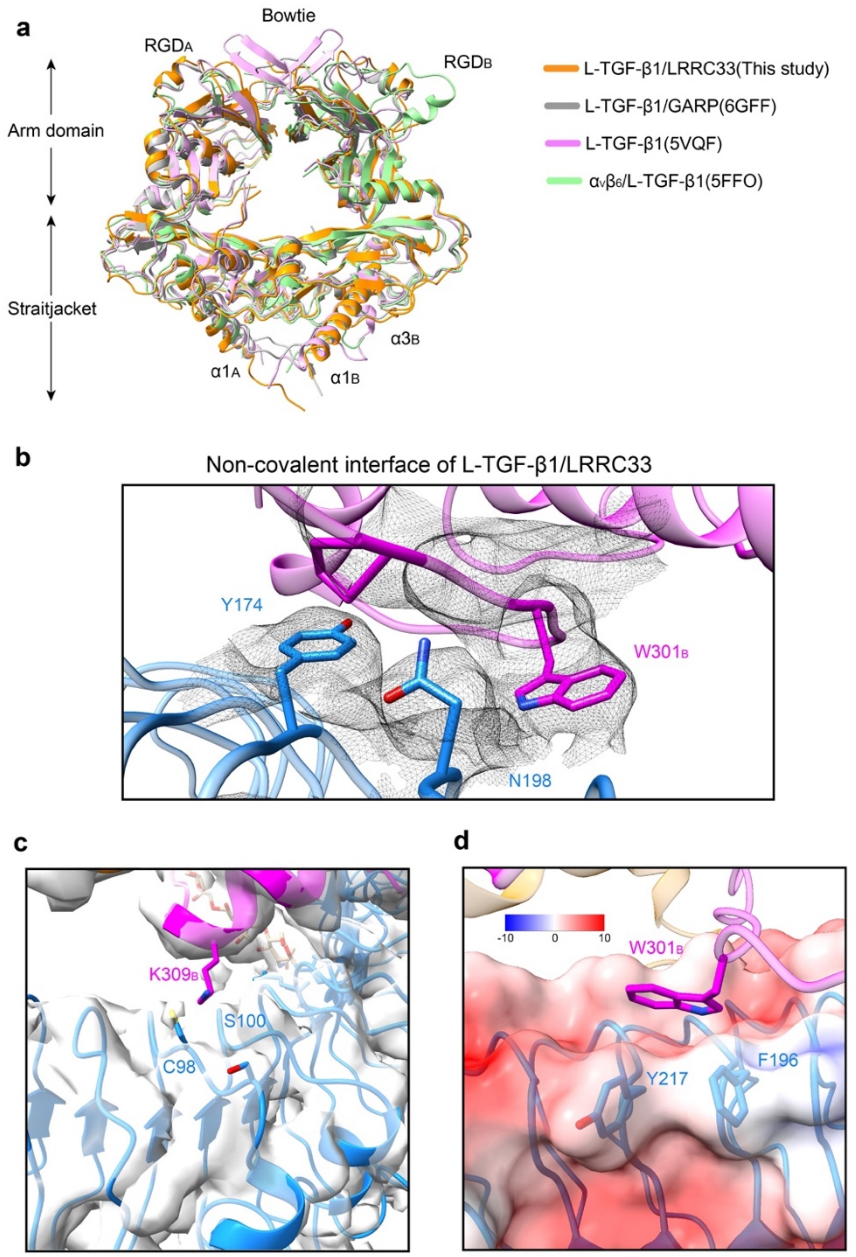

Supplementary Fig. 3 Structural analyses of the L-TGF-β1/LRRC33 complex.

a Comparison of the L-TGF-β1 structure with the reported ones. **b** Cryo-EM densities of the core non-covalent interface between L-TGF-β1 and LRRC33. The contour level is 0.202. The side chains of Trp301 on L-TGF-β1, and Tyr174 and Asn198 on LRRC33 are shown. **c** Cryo-EM densities of the marginal non-covalent interface between L-TGF-β1 and LRRC33. The contour level is 0.251. The side chains of Lys309 on L-TGF-β1, and Cys98 and Ser100 on LRRC33 are shown. **d** Surface electron potential (in units of $k_B T/e$, where k_B is the Boltzmann constant, T is the absolute temperature, and e is the elementary charge) of the L-TGF-β1 Trp301 binding pocket on LRRC33, as calculated at pH 7.0 and 0.15 M concentrations of monovalent cations and anions.

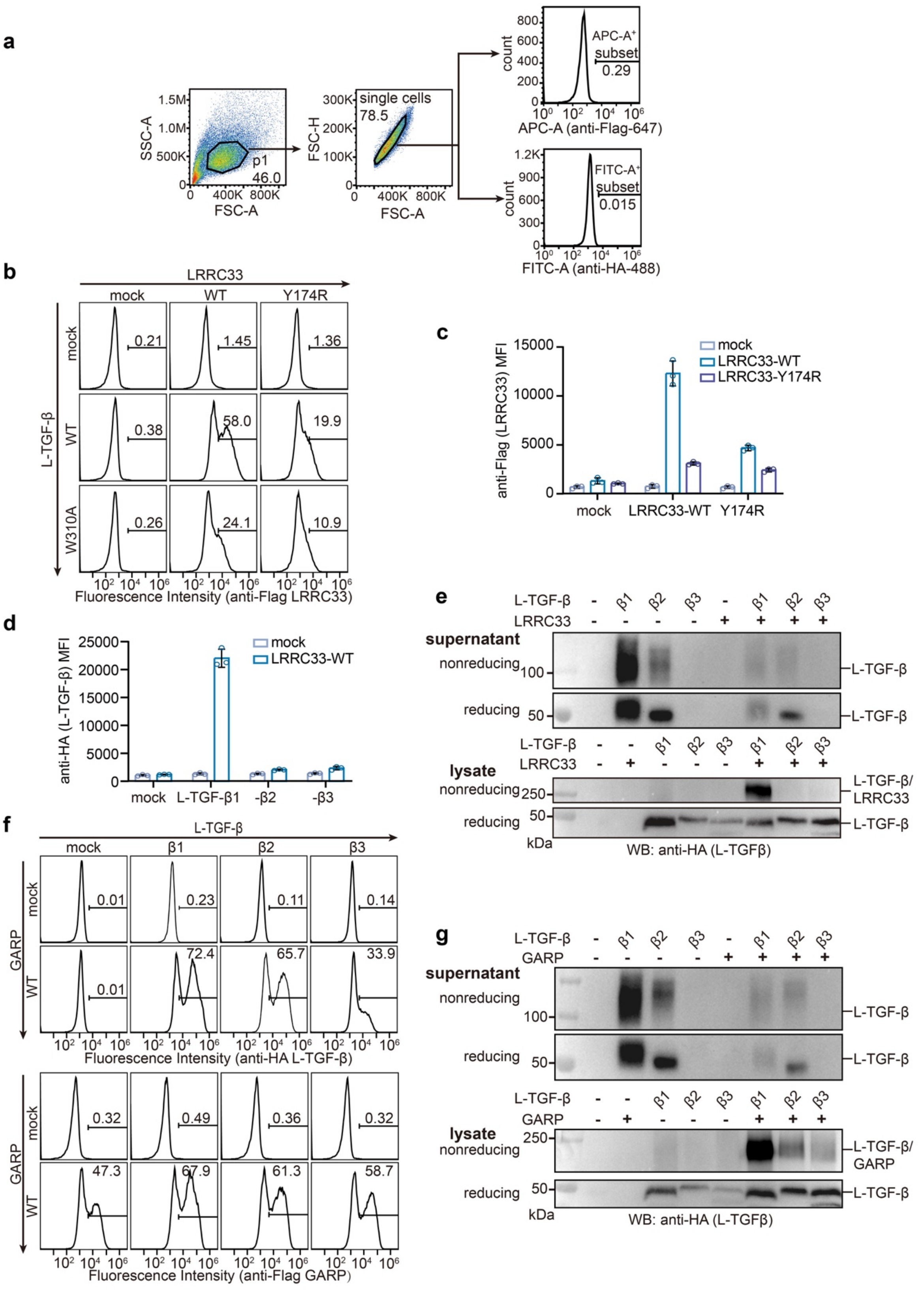
a
SSC-A
FSC-A
p1
46.0
FSC-H
single cells
78.5
APC-A+ subset
0.29
APC-A (anti-Flag-647)
FITC-A+ subset
0.015
FITC-A (anti-HA-488)
count
b
LRRC33
mock
WT
Y174R
L-TGF-β
W310A
Fluorescence Intensity (anti-Flag LRRC33)
c
anti-Flag (LRRC33) MFI
mock
LRRC33-WT
LRRC33-Y174R
d
anti-HA (L-TGF-β) MFI
L-TGF-β1
-β2
-β3
e
L-TGF-β
LRRC33
supernatant
nonreducing
reducing
lysate
L-TGF-β/ LRRC33
kDa
WB: anti-HA (L-TGFβ)
f
GARP
Fluorescence Intensity (anti-HA L-TGF-β)
Fluorescence Intensity (anti-Flag GARP)
g
L-TGF-β/ GARP

Supplementary Fig. 4 Surface presentation of different L-TGF-β variants by LRRC33 and/or GARP.

a Representative flow cytometry gating strategy for cell surface presentation of LRRC33, GARP, and L-TGF-β. **b** Surface expression of LRRC33 on transfected Expi293F cells were measured by flow cytometry (anti-Flag). Number in each histogram indicates the percentage of LRRC33+ (anti-Flag+) subset. **c** MFI (Mean Fluorescence Intensity) of LRRC33 (anti-Flag) on differently transfected Expi293F cells. All experiments were done in triplicate (n = 3 biologically independent experiments, mean ± s.d.). **d** MFI (Mean Fluorescence Intensity) of cell surface L-TGF-β isoforms (anti-HA). All experiments were done in triplicate (n = 3 biologically independent experiments, mean ± s.d.). **e** Culture supernatants and total lysates of Expi293F cells transfected with indicated plasmids were subjected to non-reducing and reducing western blot analyses with anti-HA antibody (for L-TGF-β). The experiment was repeated three times independently with similar results. **f** Surface presentation of L-TGF-β1, -β2, and -β3 and GARP measured by flow cytometry (anti-HA for L-TGF-β staining, and anti-Flag for GARP staining). **g** Culture supernatants and total lysates of Expi293F cells transfected with indicated plasmids were subjected to non-reducing and reducing western blot analyses with anti-HA antibody (for L-TGF-β). The experiment was repeated three times independently with similar results. Source data are provided as a Source Data file.

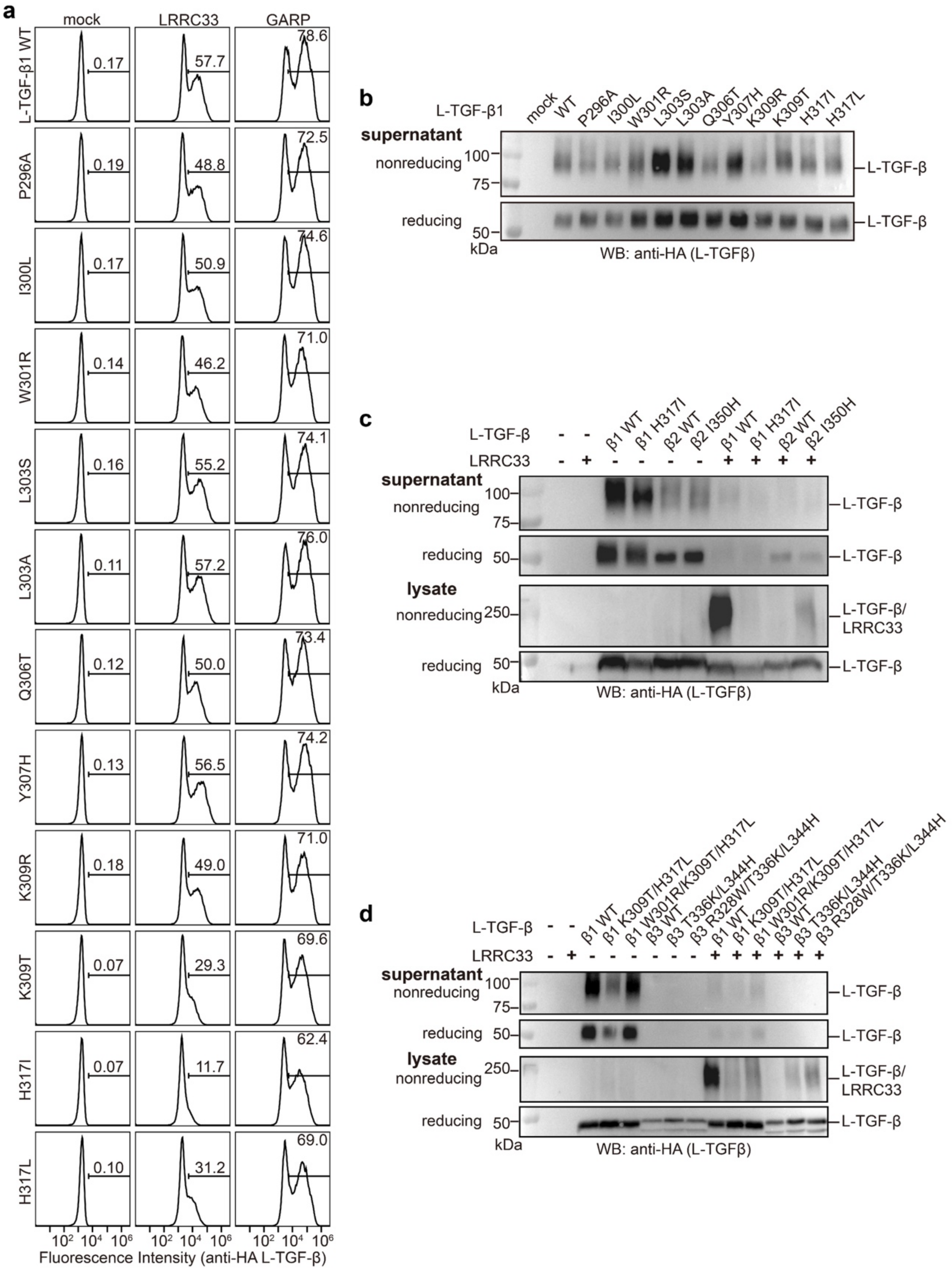

Supplementary Fig. 5 Mapping for specificity determining residues on L-TGF-β1 for LRRC33 complex formation.

a Surface expression of L-TGF-β1 single mutations by LRRC33 and GARP measured by flow cytometry (anti-HA). Number in each histogram indicates the percentage of L-TGF-β1+ (anti-

HA+) subset. **b** Culture supernatants of Expi293F cells transfected with indicated plasmids were subjected to non-reducing and reducing western blot analyses with anti-HA antibody (for L-TGF-β). The experiment was repeated three times independently with similar results. **c-d** Culture supernatants and total lysates of Expi293F cells transfected with indicated plasmids were subjected to non-reducing and reducing western blot analyses with anti-HA antibody (for L-TGF-β). The experiment was repeated three times independently with similar results. Source data are provided as a Source Data file.

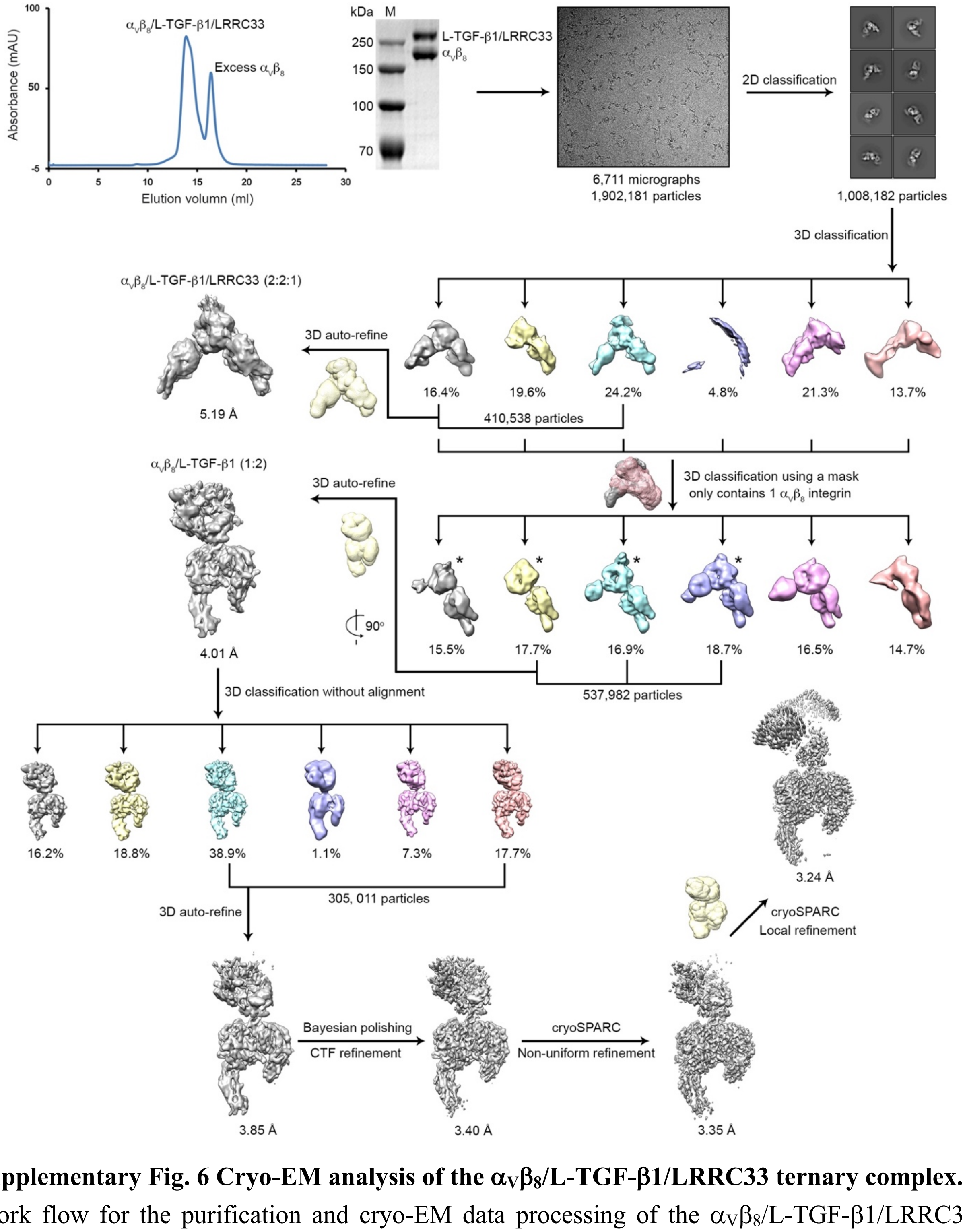

Supplementary Fig. 6 Cryo-EM analysis of the $\alpha_V\beta_8$/L-TGF-β1/LRRC33 ternary complex. Work flow for the purification and cryo-EM data processing of the $\alpha_V\beta_8$/L-TGF-β1/LRRC33 complex. The 2:2:1 $\alpha_V\beta_8$/L-TGF-β1/LRRC33 complex was refined to medium-resolution (5.19 Å), whereas the 1:2 $\alpha_V\beta_8$/L-TGF-β1 complex was refined to high-resolution (3.24 Å). The size exclusion chromatography (SEC) experiment was repeated three times independently with similar results. Source data are provided as a Source Data file.

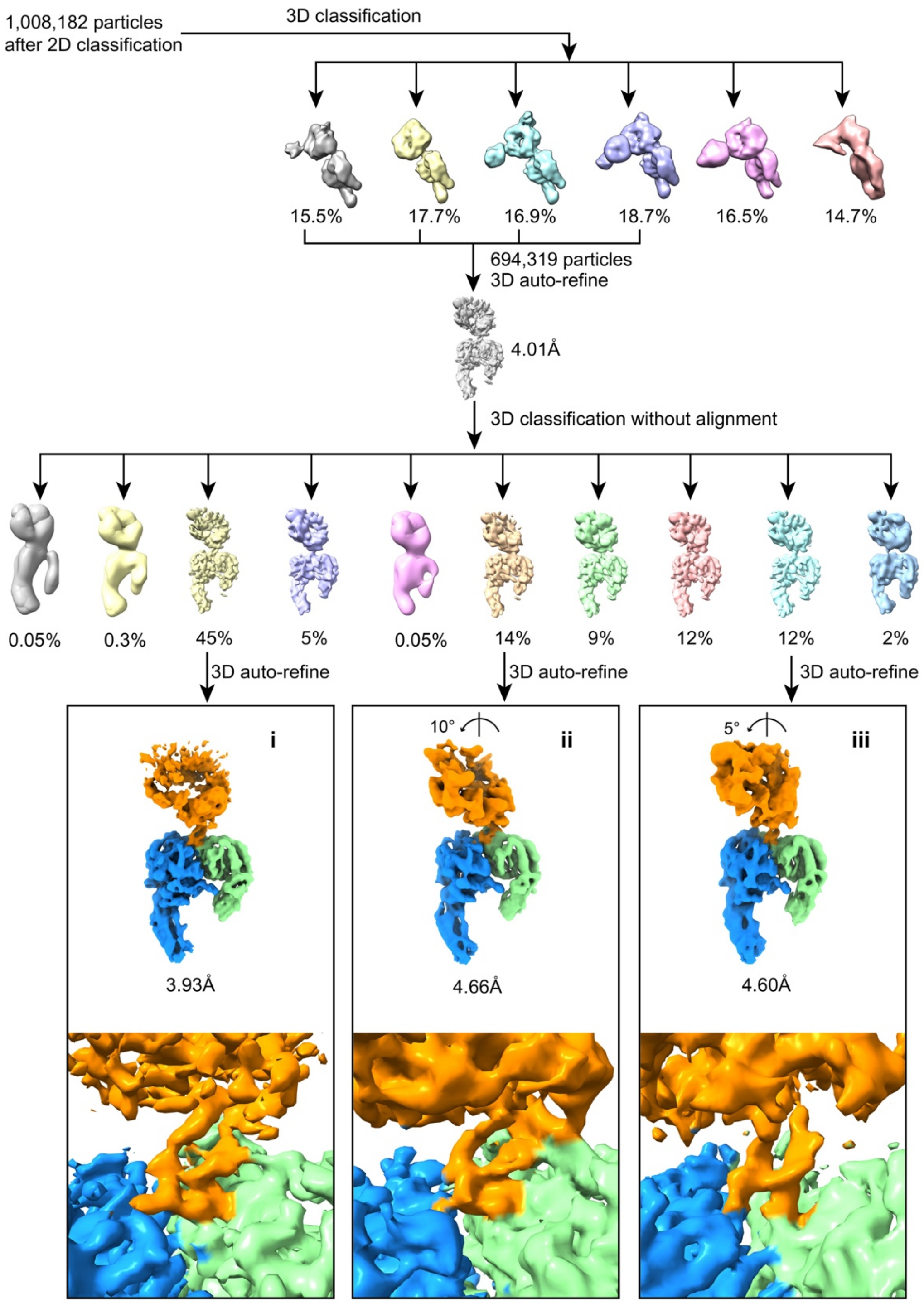

Supplementary Fig. 7 Structural plasticity analysis of the $\alpha_V\beta_8$/L-TGF-β1 complex.

Exquisite 3D classification specific to the 1:2 $\alpha_V\beta_8$/L-TGF-β1 complex in our dataset. The EM densities of L-TGF-β1 and the relative position between $\alpha_V\beta_8$ and L-TGF-β1 varied considerably between different subclasses, especially for the three classes with relatively higher resolution.

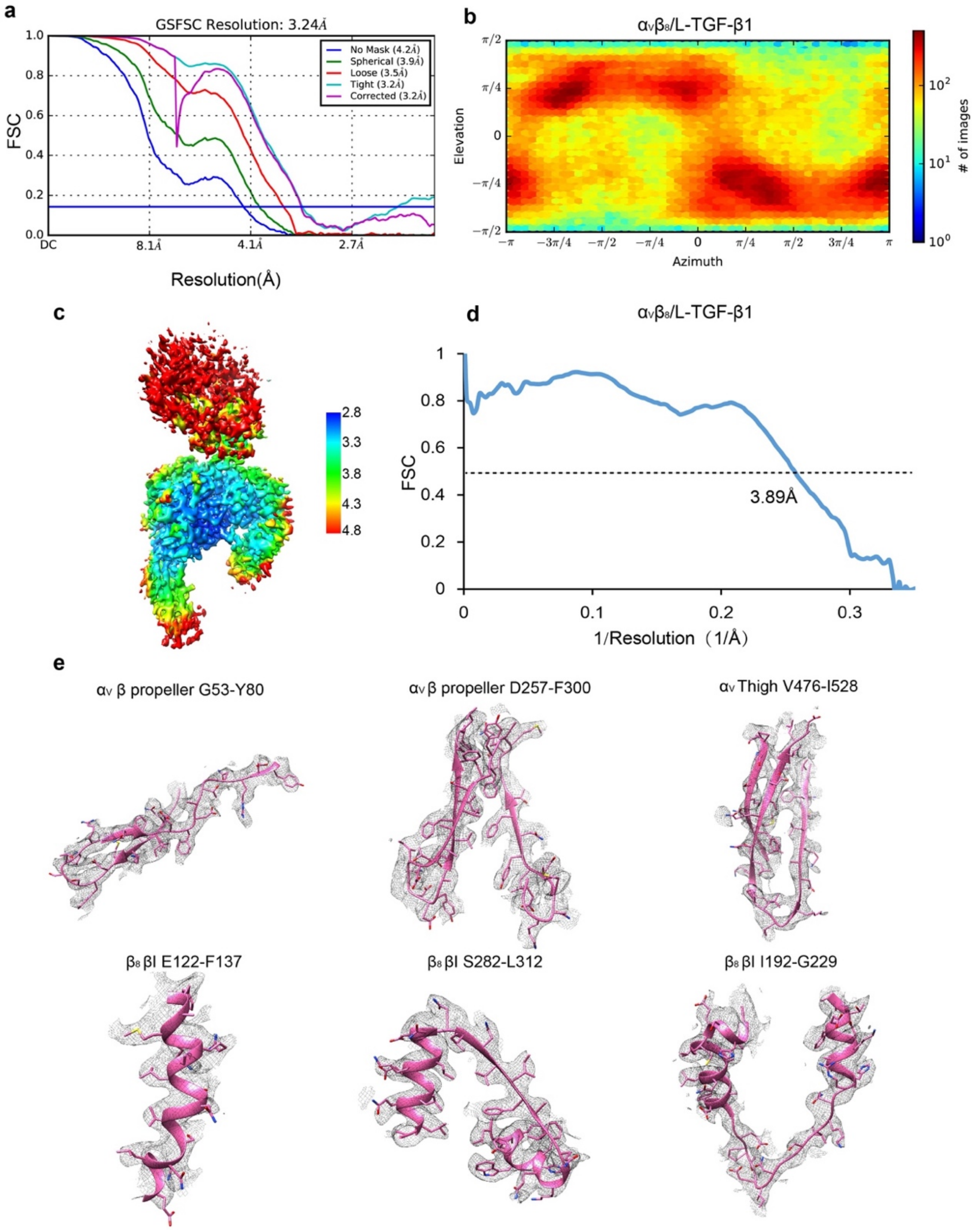

Supplementary Fig. 8 Assessment of the cryo-EM data and structural model for the $\alpha_V\beta_8$/L-TGF-β1 complex.

a Fourier Shell Correlation (FSC) curves between reconstructions of two half-datasets. **b** Angular distributions of all the particles used for reconstruction. **c** Local resolution estimation of the cryo-EM map. The contour level is 0.223. **d** FSC curve between the refined structure and the cryo-EM map. **e** Representative cryo-EM densities of different regions. The contour level is 0.703.

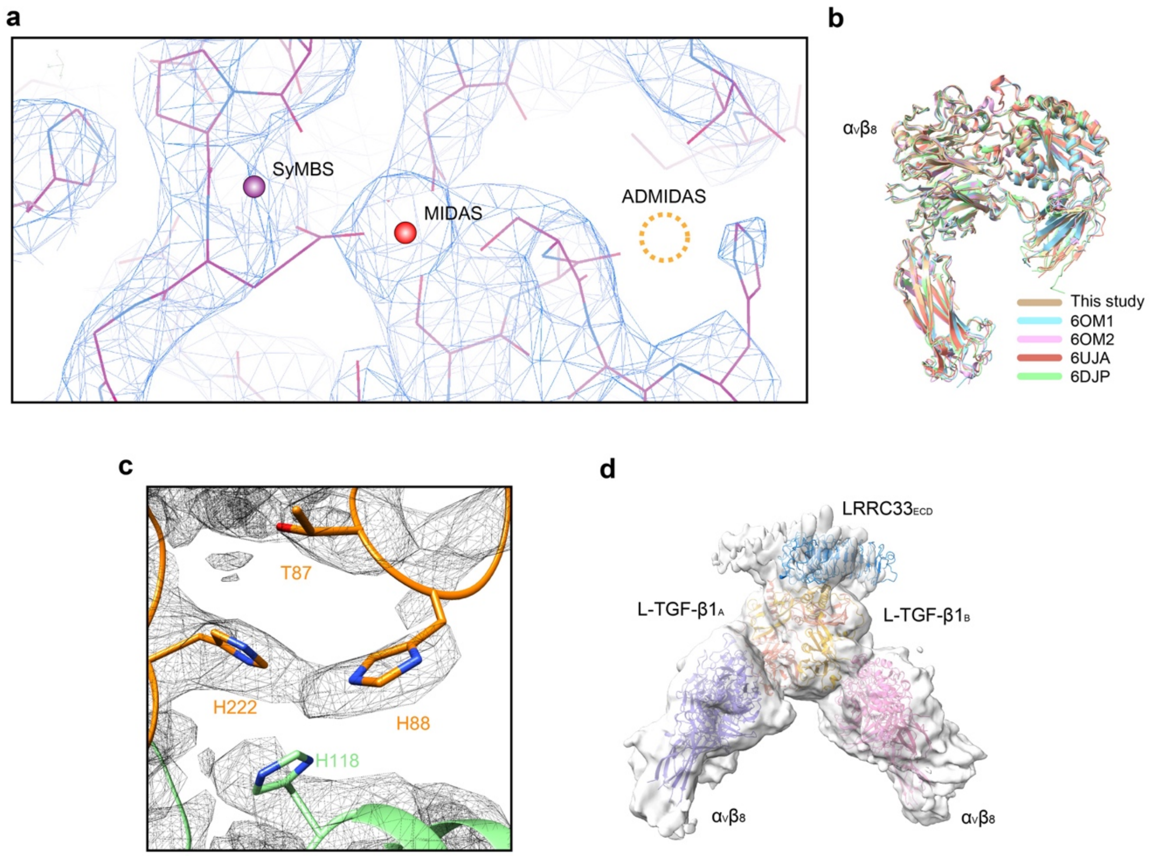

Supplementary Fig. 9 Structural analyses of the $\alpha_V\beta_8$/L-TGF-β1/LRRC33 complex.
a Metal ion is absent in the ADMIDAS (dashed circle). Cryo-EM densities around the MIDAS (red) and SyMBS (purple) cations are shown as blue meshes. The contour level is 0.704. The adjacent residues are shown as sticks. **b** Superposition of currently reported integrin $\alpha_V\beta_8$ structures. 6DJP and 6OM1: $\alpha_V\beta_8$ alone; 6OM2: $\alpha_V\beta_8$ in complex with L-TGF-β1 ligand peptide; 6UJA: $\alpha_V\beta_8$ in complex with porcine L-TGF-β1. **c** Cryo-EM densities around Thr87, His88, and His222 of L-TGF-β1 and His118 of integrin β_8 subunit, are shown as black meshes. The contour level is 0.703. L-TGF-β1 and integrin β_8 are colored in orange and green, respectively. **d** Assembly of the 2:2:1 $\alpha_V\beta_8$/L-TGF-β1/LRRC33 structure model by fitting the obtained structures of $\alpha_V\beta_8$/L-TGF-β1 and L-TGF-β1/LRRC33 into the medium-resolution cryo-EM map in Supplementary Fig. 6. The structures are shown in ribbon presentation and the EM map is shown as semitransparent surface. The contour level of the map is 0.006.

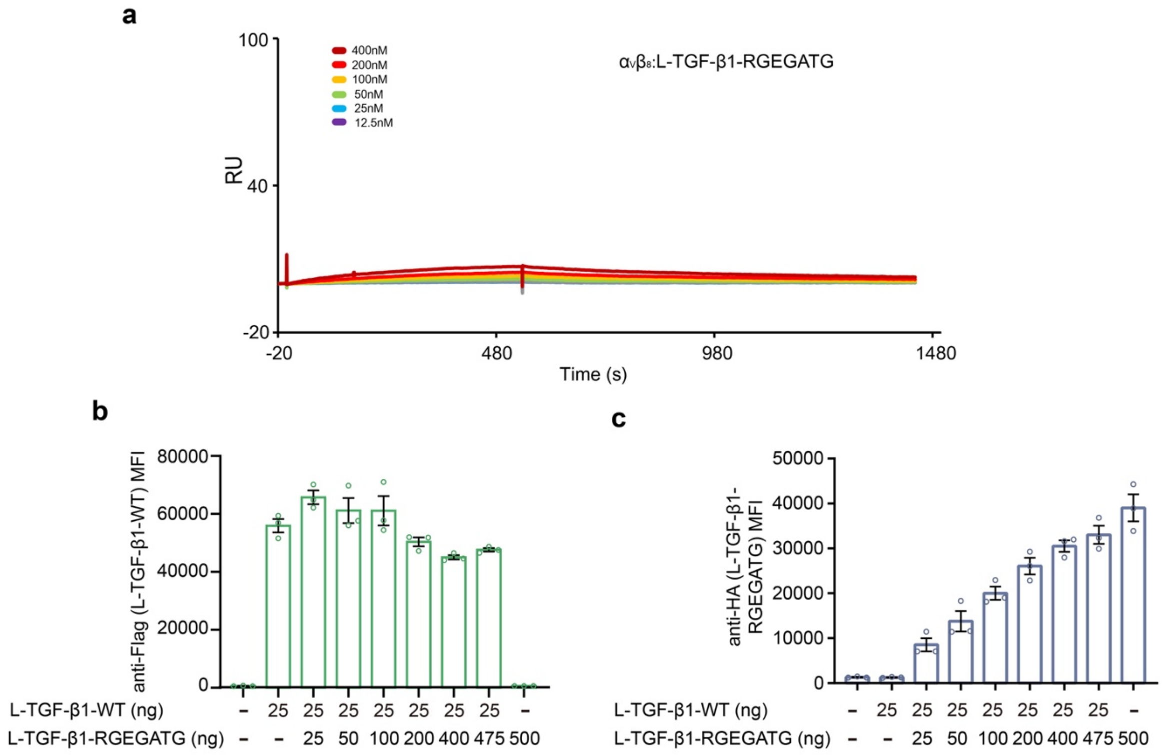

Supplementary Fig. 10 Functional validation of the 2:2 $\alpha_V\beta_8$/L-TGF-β1 binding model.
a SPR result showing the L-TGF-β1-RGEGATG variant cannot bind integrin $\alpha_V\beta_8$. **b-c** Surface expression levels of L-TGF-β1 in cells with different transfected ratios of WT and mutated constructs (calibrated by the MFI values). All experiments were done in triplicate (n = 3 biologically independent experiments, mean ± s.d.). Source data are provided as a Source Data file.

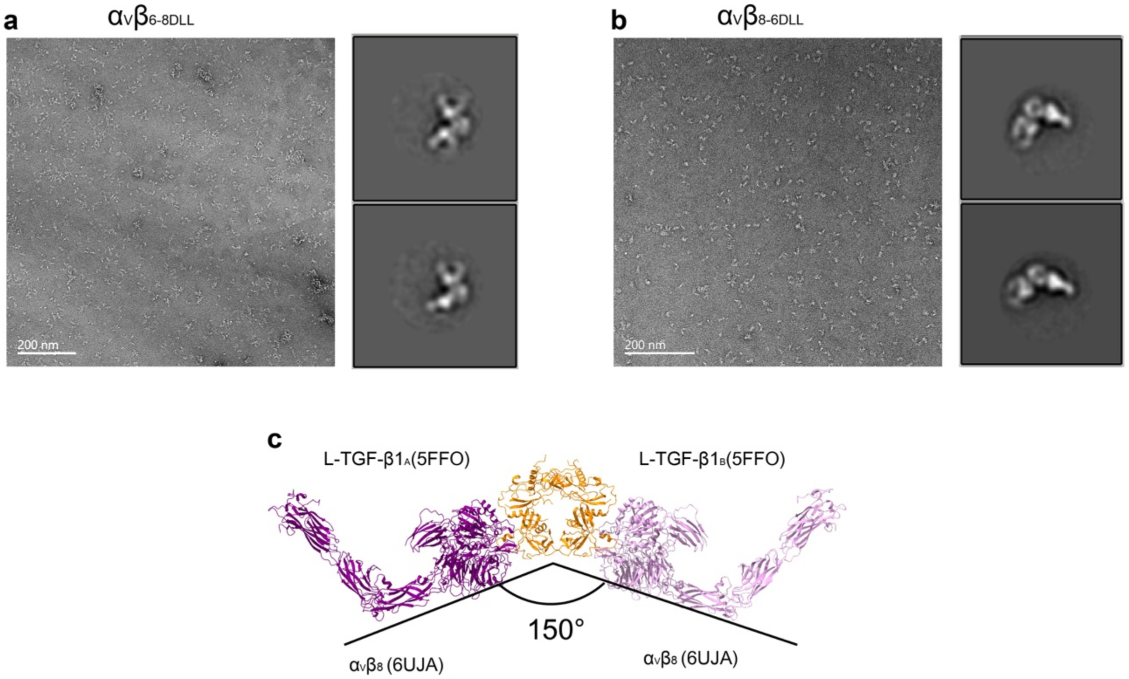

Supplementary Fig. 11 Structural analyses of different integrin/L-TGF-β1 complexes.

a The representative raw micrograph and 2D classes of the $\alpha_V\beta_{6\text{-}8DLL}$/L-TGF-β1 complex**.** $\alpha_V\beta_{6\text{-}8DLL}$ adopts an open conformation. 22 micrographs were collected and processed for this dataset. **b** The representative raw micrograph and 2D classes of the $\alpha_V\beta_{8\text{-}6DLL}$/L-TGF-β1 complex. $\alpha_V\beta_{8\text{-}6DLL}$ adopts a closed conformation. 70 micrographs were collected and processed for this dataset. **c** A modeled structure of the 2:2 $\alpha_V\beta_8$/L-TGF-β1 complex based on the symmetric operation of the reported 1:2 $\alpha_V\beta_8$/L-TGF-β1 structure (PDB code: 6UJA). The intact L-TGF-β1 structure is derived from that in the 1:2 $\alpha_V\beta_6$/L-TGF-β1 complex (PDB code: 5FFO). The lower leg of integrin α_V (PDB code: 6AVU) is integrated into this model.

Supplementary Table 1. Cryo-EM data collection, refinement and validation statistics.

	L-TGF-β1/LRRC33 (EMDB-33571) (PDB 7Y1R)	$\alpha_V\beta_8$/L-TGF-β1 (EMDB-33572) (PDB 7Y1T)	$\alpha_V\beta_8$/L-TGF-β1/LRRC33 (EMDB-33573)
**Data collection and processing**			
Magnification	130,000	130,000	130,000
Voltage (kV)	300	300	300
Electron exposure (e^-/Å^2)	57.5	57.5	57.5
Defocus range (μm)	1.0-3.0	1.0-3.0	1.0-3.0
Pixel size (Å)	1.055	1.055	1.055
Symmetry imposed	C1	C1	C1
Initial particle images (no.)	1,250,966	1,902,181	1,902,181
Final particle images (no.)	254,673	305,011	410,538
Map resolution (Å)	4.01	3.24	5.19
FSC threshold	0.143	0.143	0.143
Map resolution range (Å)	3.4-14	2.8-12	4.2-21.3
**Refinement**			
Initial model used (PDB code)	6GFF	6UJA	
Model resolution (Å)	4.82	3.89	
FSC threshold	0.5	0.5	
Model resolution range (Å)	-	-	
Map sharpening *B* factor (Å^2)	-	64.6	
Model composition			
Non-hydrogen atoms	8,565	8,112	
Protein residues	1,041	1,030	
Ligands	-	-	
B factors (Å^2)			
Protein	77.88	82.93	
Ligand	-	-	
R.m.s. deviations			
Bond lengths (Å)	0.002	0.002	
Bond angles (°)	0.619	0.518	
Validation			
MolProbity score	1.86	1.73	
Clashscore	8.73	8.09	
Poor rotamers (%)	0.00	0.00	
Ramachandran plot			
Favored (%)	94.20	95.86	
Allowed (%)	5.80	4.14	
Disallowed (%)	0.00	0.00	
